# Numerical simulation analysis of pile-soil interaction under earthquake action

**DOI:** 10.1371/journal.pone.0312689

**Published:** 2025-03-10

**Authors:** Yifei Wang, Zhanfang Huang, Ruixue Hu, Lichao Bai, Junjie Zheng, Yi Chen, Xiaohong Bai

**Affiliations:** 1 School of Civil Engineering and Geomatics, Shandong University of Technology, Zibo, China; 2 Key Laboratory of Highway Construction & Maintenance Technology in Loess Region, Shanxi Transportation Research Institute, Taiyuan, China; 3 School of Civil Engineering, Wuhan University, Wuhan, China; 4 College of Civil Engineering, Taiyuan University of Technology, Taiyuan, China; Guizhou University, CHINA

## Abstract

Pile foundation is a commonly recognized form of foundation, and earthquakes are a common seismic damage phenomenon. Accidents resulting from reduction in pile bearing capacity due to earthquakes pose a great threat to people’s lives and safety. This article investigates the interaction between soil and piles under earthquake action. Utilizing the MIDAS GTS NX finite element software, the vertical bearing characteristics of piles under earthquake action are studied. Obtained acceleration of piles, pile settlement, pile axial force, pile top horizontal displacement, soil pore water pressure, and pore pressure ratio under different earthquake magnitudes. The research results indicate that as the depth increases, the acceleration at the pile top is significantly greater than that at the pile bottom, with an average increase of 20% in acceleration at three different earthquake magnitudes; Both the beginning of the pore pressure ratio growth and the ultimate reaching of its stable pore pressure ratio coincide with a rise in earthquake magnitude. Additionally, the axial force of the pile body also increases with the magnitude of the earthquake, and the maximum axial force of the pile body can increase by 40% at the same time. Simultaneously, the magnitude of the earthquake influences both the displacement of the pile body and the settling of the pile top. This article can provide reference for pile foundation design and engineering construction in liquefaction sites.

## 1. Introduction

In the past several years, as earthquakes occur frequently, it is very prevalent that non-cohesive foundations, like chalky and sandy soils, lose their load-bearing capacity under seismic action, leading to structural damage, which poses safety hazards to people’s lives and property safety [1]. The cause of the accident is that saturated sand loses its shear strength due to strong vibration under earthquakes, dynamic loads, or other external forces, causing the sand particles to be in a suspended state and resulting in foundation failure [2]. Most structural failures are attributed to a reduction in foundation bearing capacity [3]. And accidents caused by a reduction in foundation bearing capacity are one of the reasons, which makes it necessary to study the dynamic response between piles and soil [4,5].

The dynamic viscosity of liquefied soil was measured by Sawicki A [6], who also demonstrated that liquefied soil may be conceptualized as a viscous fluid. In addition, a viscous fluid model was developed and the experimental values were found to agree with some theoretical estimates. Fattah, M. Y et al. [7] conducted response tests on a series of pile groups with different relative densities of soil under dynamic loads. The results showed that the dynamic response mechanism of piles and soil under dynamic horizontal vibration includes not only vertical and horizontal displacement, end bearing capacity, peak acceleration, and foundation peak velocity, but also the variation and distribution of acceleration over time under different soil states. It is believed that seismic waves have damping effects or attenuation, and the amplitude increases with the relative density of the soil. Maheshwari B [8] based on equivalent linearisation with hyperbolic model of soil. He proposed a method that would couple the pile to the soil to get over the material nonlinearity of the soil and the geometric nonlinearity caused by the separation. Dynamic response of soil-pile systems was found to increase and the dynamic stiffness was found to decrease significantly. Krim A [9] carried out loading tests on mixtures of recycled sand-clay with low clay content at different perimeter pressures with the help of triaxial loading device. After examining the impact of grading features and clay content on liquefaction resistance, it was discovered that the drainage shear strength of sand-clay mixtures is limited due to their grading properties. The grading features of sand-clay mixes determine their undrained shear strength, and a 15% rise in clay content reduces the mixtures’ resistance to liquefaction. Fattah, M. Y et al. [10] used a vibration box to simulate the seismic motion caused by the foundation and pile groups, in order to measure the vertical and horizontal displacement response of pile groups under dynamic loads. It was found that due to the densification of soil during the vibration process, an increase in vibration frequency leads to a decrease in the oscillation of wave propagation values. As the pile spacing ratio s/d increases, the internal force slightly decreases. L. M [11] concluded that liquefaction may also occur in unsaturated soils and investigated the liquefaction discrimination method for unsaturated sandy soils via performing cyclic triaxial experiments on various sandy soils, the findings demonstrated a strong association between apparent viscosity and strain liquefaction triggering criterion, confirming the validity of apparent viscosity as a liquefaction triggering measure. S. Bhattacharya [12] found that under horizontal loading, the pile foundations will have a reduced vertical bearing capacity. The reduction in vertical load diminishes with the rise in horizontal load. Fattah, M. Y et al. [13] studied the dynamic response of a single pile in dry saturated sand under relative rotating machine excitation by creating a small-scale physical model. The axial strain along the pile increased with the increase of operating frequency. The vibration amplitude at the pile head also increases with the increase of operating frequency. According to Saeedi [14], as the pile, relative stiffness of the pile-soil, and relative density of the soil all grow, so does the pile’s largest bending moment. Fattah, M. Y et al. [15] conducted experiments on the excess pore water pressure generated around a single pile and a group of piles excited by two relatively rotating machines embedded in saturated sand by creating a small physical model. They found that the excess pore water pressure was affected by the aspect ratio, length, spacing, and number of piles. However, for a single pile, at the same frequency, the amplitude of vertical vibration decreased with an increase in pile length.

In numerical modelling studies, Tang L [16] Vibrating table experiments on the embedding behavior of reinforced concrete low-cap pile groups in a sandy soil. The properties of reinforced concrete low bearing piles buried in such formations has been investigated. Tang L [17] Vibrating table experiments on the embedment behavior of a cluster of reinforced concrete low cap piles in a sandy soil. The properties of reinforced concrete low bearing piles embedded in this type of ground were investigated. In addition, numerical simulation analyses were carried out. The law that kinematic effects are largely associated with both sand permeability and *EI* (Pile stiffness, pile stiffness refers to its ability to resist deformation caused by external forces.) was obtained. Bhatnagar S [18] carried out numerical modelling of embankments built on loose liquefiable sediments. By studying the parameters such as displacement, liquefaction tendency and superporous pressure, it was found that ground displacement due to seismic liquefaction is the primary reason for damage to soil structures composed of or under loose saturated granular soils. S. Mohammad [19] used the OpenSees finite element method to model the complex soil structure single pile interaction and soil liquefaction, taking into account bidirectional seismic loads and the influence of environmental loads on the structure. He studied the seismic performance of offshore wind turbine (OWT) foundations and found that the seismic response of the single pile structure system would intensify under the action of environmental and seismic loads. However, increasing the size of a single pile does not necessarily improve the seismic performance of the foundation [20]. It is investigated if numerical methods can forecast embankment deformation caused by earthquakes. Software for numerical simulation provides a qualitative prediction of the vertical settlement and pore pressure of embankment. Pathak RS [21] proposed the Basic Empirical liquefaction model (EELM) based on dynamic response, and studied the evaluation method of soil liquefaction potential (LP) by associating the most significant parameters that reflect the dynamic response of the soil to actual field behavior. It is found that EELM method can be used to evaluate soil liquefaction potential (LP). Zhang Yongsheng [22] established numerical models of rubble piles under different working conditions and conducted seismic response analysis. It is found that gravel pile has good drainage effect and remarkable liquefaction resistance in the range of triple pile diameter. Yu Lei [23] carried out numerical calculation and analysis on the proposed seismic simulation vibration test model. It was observed that when the mass of the superstructure is greater, the bending moment of the pile-earth structure is also greater. Yoo B [24] established an improved soil-pile interface model. It was studied the downward-moving lateral displacement of liquefied earth. Liquefaction of inclined ground will result in bending moment and permanent deformation in the pile. The rule that the inclination angle of the slope affects the sliding quality, resulting in elevated bending moments in the pile with a larger inclination angle is obtained. Utilizing a 3D effective stress finite element (FE) analysis for modeling, Tang L [25] carried out a vibration table test on pile foundations in liquefiable soil made up of overlaying soft clay and liquefiable sand. The reinforcement of liquefiable soils by pile foundations in liquefiable soils consisting of overlying soft soils and liquefiable sandy soils was investigated, and it was found that pile foundations have a significant pinning effect, which can reduce lateral soil displacement. The piles’ lateral deformation is lessened by the larger pile diameter along with fixed pile head limits. Pan Xiaoguang [26] analyzed the bearing deformation of rigid grid small pile composite foundation based on Midas GTS. It was found that rigid small pile foundation can remarkably enhance the foundation bearing capacity, decline the foundation settlement deformation, and improve the bearing capacity of the foundation. According to the limit analysis theorem, Di Laora [27] suggested a novel formulation of the interaction diagram that would provide the axial bearing capacity on the outermost pile as the eventual state of the pile group. Huang Yu et al. [28] established the cyclic plasticity of saturated liquefied sand and found that the soil around the pile soil structure in a liquefaction site is more prone to liquefaction due to the influence of dynamic loads compared to the soil at the identical depth in a free site. Wu Yuedong [29] developed a negative and positive skin friction model for pile foundations in elastic and plastic states and derived a closed-form analytical solution for calculating the lateral friction resistance of pile foundations in elastic and plastic states.

In summary, numerous domestic and foreign scholars have carried out in-depth studies on the deformation of soils and piles under seismic effects, which constitutes a crucial area of study for earthquake engineering, including pile foundation instability, dynamic response of soil, and structural seismic design and reinforcement. However, relatively few studies have been conducted on pile-soil interactions, so further in-depth research is needed. In this paper, we mainly investigate the dynamic response of pile-soil system under seismic action, with emphasis on the vertical bearing properties of pile-soil system under seismic action. The vertical bearing properties of piles under seismic action are investigated by means of MIDAS GTS NX FE software, and the horizontal displacement at the top of the pile, axial force of the pile body, the trends of pile settlement, pile acceleration, pore pressure ratio together with pore water pressure of the soil are derived under different earthquake magnitudes. This reveals the impact mechanism of earthquake action on pile foundations, and also demonstrates the extent to which various seismic magnitudes affect the bearing performance of the piles. This article provides an important basis for a deeper understanding of earthquake phenomena and pile foundation engineering design, and provides scientific references for improving earthquake disaster prevention capabilities and people’s disaster resistance capabilities.

## 2 Establishment of 3D numerical model

### 2.1 Numerical modelling and selection of calculation parameters

**Fig 1:**
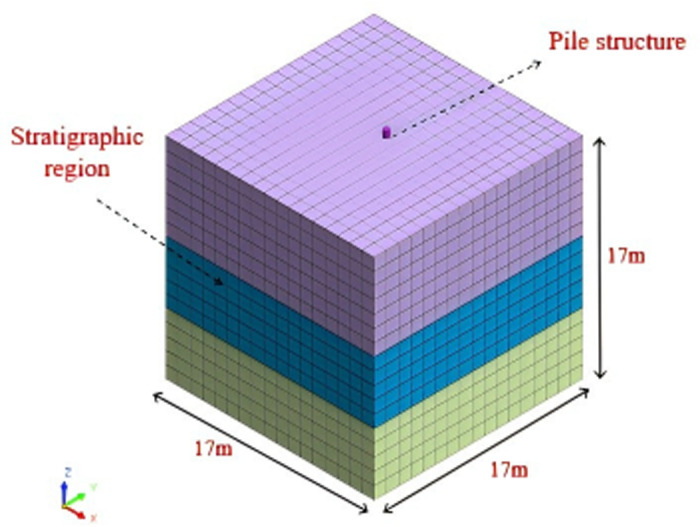
Three dimensional numerical model of single pile earthquake.

MIDAS GTS NX is a general purpose numerical analysis software [30]. It is a multifunctional software for stress and strain analyses, stability analyses and seepage analyses during the construction phase of tunnels and pits. For Fig 1 of this model, MIDAS finite element simulation software was used to implement the modelling as well as the result extraction process. In order to give preliminary shape to the model, modelling and geometric construction are carried out firstly. Then, the model grid was divided into 4899 grids and 6405 nodes, with a total of 1994 grids and 2500 nodes for sandy soil; The loess is divided into 1445 grids and 1944 nodes; The cohesive soil is divided into 1445 grids and 1944 nodes,; The pile body is divided into 15 grids and 17 nodes. This step determines the accuracy of the model and the efficiency of the calculation [31,32]; The parameters of the material are then defined determining the external loads and constraints to which the model is subjected. The model in this paper is modelled using dynamic loading. Besides, the FE method is applied to solve the model and calculate the response of the pile-soil system in terms of stresses, displacements and deformations. Finally, the model generates the results of the solution analysis.

This model accurately simulated the stress, deformation, pore pressure ratio as well as pore water pressure of individual pile foundation under different earthquake magnitudes and time conditions, and conducted comparative analysis. The model is composed of two primary components, the pile body and the soil surrounding the pile. Based on the basic overview of the overall construction given, the model length is 17m, width is 17m, and height is 17m. The parameters of the pile foundation are referred to in reference [33], and the soil layer parameters are chosen [34,35] as presented in Table 1. The soil is simulated using 3D solid elements, and the pile structure is simulated using 1D rod elements. Under on-site working conditions, the soil stratification and properties are more complex and variable, making it difficult to qualitatively stratify. Due to the consideration of finite element calculation, the model is set up with three soil layers, and the finite element numerical calculation considers the soil as a continuous, uniform, and isotropic medium. A general overview of the model is illustrated in Fig 1.

**Table 1:** Model parameter selection.

Soil type	Thickness (m)	Porosity ratio	Elastic modulus (kN/m^2^)	Bulk density (kN/m^2^)	Poisson ratio	Cohesive force (kN/m^2^)	Saturated bulk density (kN/m^3^)	Friction angle(°)
Sand	7	0.5	20000	20.10	0.3	23	20.286	33
Silt	5	0.5	23000	20.09	0.33	26	20.384	30
Clay	5	0.6	14000	19.60	0.25	30	19.698	36

### 2.2 Soil constitutive relationship

**Fig 2:**
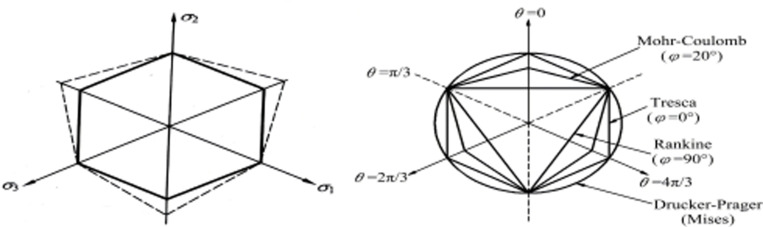
π plane Coulomb yield criterion curve.

Choosing an appropriate constitutive model is crucial for numerical simulation analysis of pile-soil interaction, ensuring the accuracy and reliability of simulation results. The model utilizes the Moore-Coulomb soil composition model, which is a desirable elasto-plastic tectonic model that is frequently employed to describe the soil’s nonlinear mechanical behavior. It combines Coulomb’s damage criterion and Hooke’s law and is extensively applied in the geotechnical engineering field. Fig 2 shows the Coulomb yield criterion curve in the π plane.

Different soils have different cohesive forces and internal friction angles, which correspond to the shear strength equation. Under the action of self weight or external force, shear stress will be generated inside the rock and soil, and as the stress increases, the strain will also increase. If it continues to develop, it will fail along a certain surface, which is called shear failure. Shear stress causes shear behavior and shear limit, i.e., shear strength. The soil shear strength is represented as follows on the basis of the Mohr-Coulomb criterion.


τ=C+σtanφ (1)

**Fig 3:**
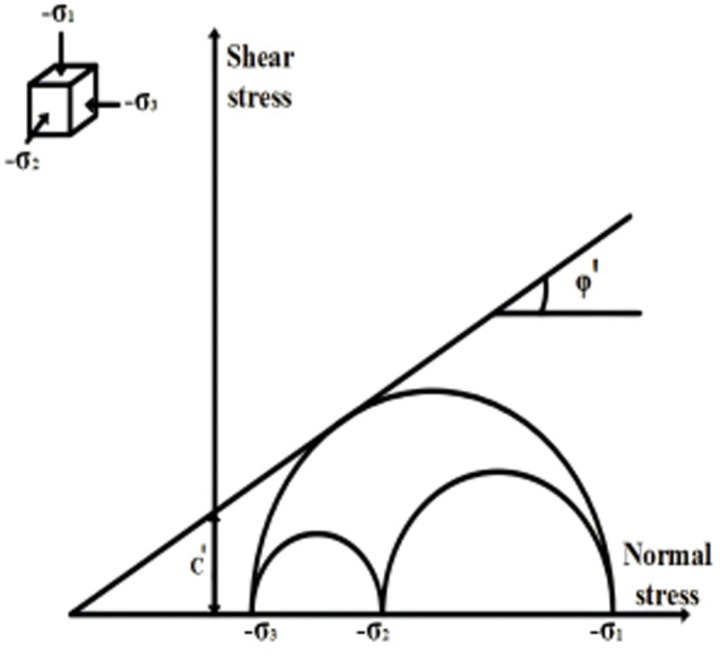
Shear Mohr Circle of Soil.

Here, *τ* denotes the soil shear strength, *C* is the soil cohesion, *σ* stands for the soil normal stress, *φ* denotes the soil internal friction angle. Mohr circle of soil shear is presented in Fig 3.

### 2.3 Interface settings

The interface model of MIDAS GTS is designed to simulate the boundary behavior of different materials or the same material. The interface model is not only applicable to geotechnical models, but also to defining various interface behaviors for the entire building and different fields. Models are often used to simulate various interfaces between rock joints or structures and soil, such as the interface between friction piles and soil, the interface between retaining walls and rock soil, and the interface between lining and rock.

This model uses an interface element defined as the contact element in the material to simulate the boundary behavior of different materials. Ensuring the requirements of finite element calculation. Interface units are not only suitable for geotechnical models, but also for defining various interface behaviors throughout the entire field of architecture and bridges. In order to better reflect the interaction between pile soil composite system under dynamic action, interface elements are applied to the pile body to couple it with the soil. The interface model follows the Coulomb friction rule and assumes that the interface friction force is directly proportional to the interfacial friction coefficient and the magnitude of the normal constraint force acting on the interface. Install a pile end unit-an embedded scaffold at the pile top, connecting the soil and pile as a whole, ensuring the convergence of the model during calculation and iteration.

### 2.4 boundary condition

In MIDAS GTS finite element simulation, the correct setting of boundary conditions is crucial for the reliability of model analysis results. Appropriate boundary condition settings can simulate the boundary behavior of real structures, thereby affecting the analysis results of stress, strain, displacement, etc. of the model.

**Fig 4:**
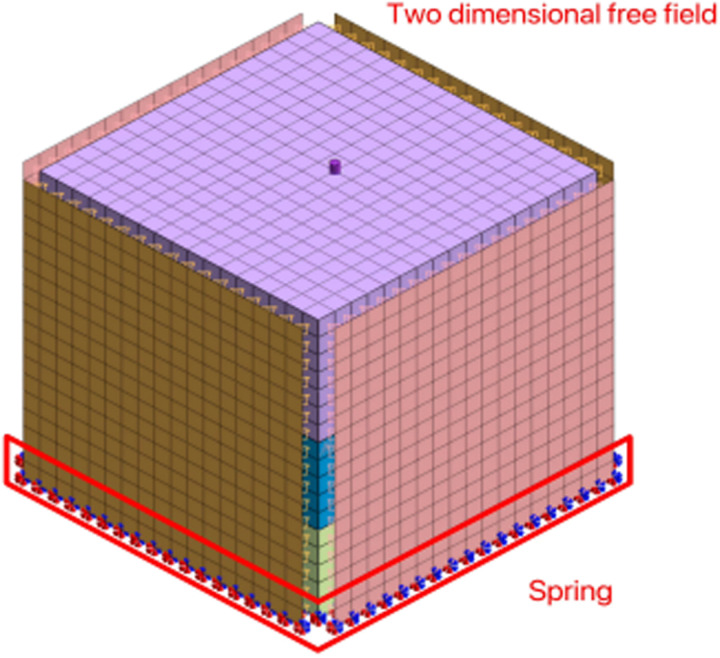
Model boundary condition.

In MIDAS GTS, springs are a commonly used boundary condition that can be used to simulate the spring support relationship between soil and external constraints. Springs can also be used to define eigenvalue analysis and response spectrum analysis. When conducting dynamic nonlinear time history analysis, eigenvalue analysis of the model is a prerequisite. When there is a support relationship between the soil and the structure, the spring can simulate the support effect of the soil on the structure. The spring damping uses the mass stiffness ratio method, with a stiffness factor of 0.615 and a stiffness damping of 0.652. Setting two-dimensional free field boundaries around the model to simulate the unconstrained behavior of soil under natural conditions can reduce the influence of boundary effects. A two-dimensional free field allows seismic waves and stresses to propagate freely at the model boundary, which can truly reflect the response of soil under seismic action. Obviously, this article studies the bearing characteristics of pile bodies and the dynamic response between piles and soil under earthquake action. Setting a fixed ground spring and two dimensional free field boundary at the bottom can ensure the structural stability the accuracy of FE calculation results. Fig 4 shows the boundary condition of this model.

### 2.5 Selection of seismic waves

The EI Centro seismic band [36] was selected for this simulation calculation, with a recorded magnitude of 6.7, a epicenter distance of 9.7 kilometers, and a duration of approximately 54 seconds. This article studies using the acceleration time history from this record as the input seismic load.

**Fig 5:**
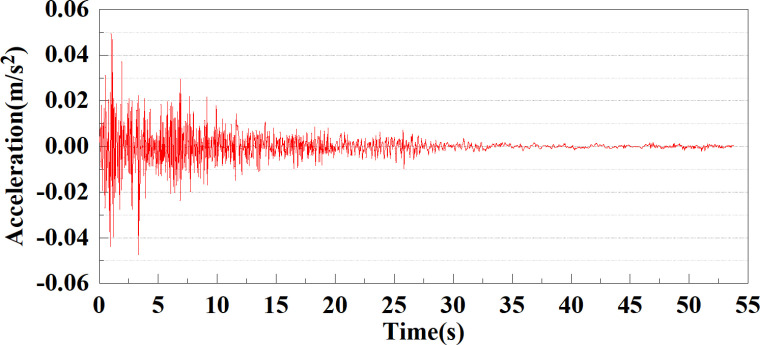
Original EI Centro seismic waves.

The energy of EI Centro waves is primarily focused in the low frequency range of 10 Hz. Due to the total duration of wave recording exceeding 54s, considering the accuracy of model calculations and time saving, only seismic waves containing the vast majority of energy can be intercepted for analysis during numerical simulation. In this study, only the first 25 seconds of seismic waves were taken for analysis. The original EL Centro seismic waves are shown in Fig 5.

## 3 Analysis of numerical simulation results for single pile earthquake

### 3.1 Analysis of pore pressure ratio and pore water pressure variations at various characteristic points with the same amplitude

**Fig 6:**
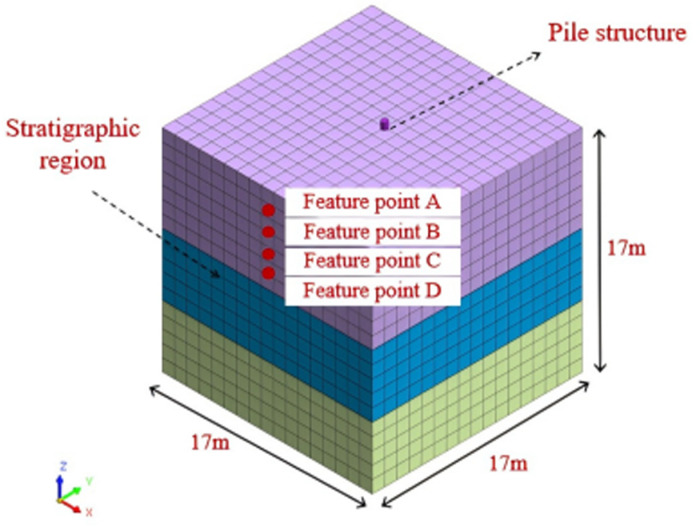
Distribution of feature points.

Divide the first layer of the single pile formation into four equally spaced blocks along Z-axis from the area of sandy soil, and take four feature points A, B, C, and D at the centerline position of each block. The distribution of feature points is shown in Fig 6.

**Fig 7:**
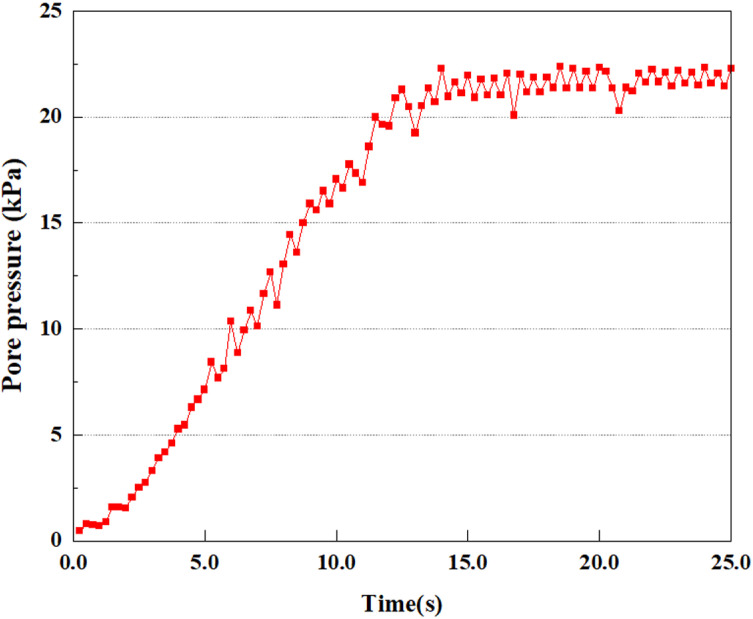
Variations in pore water pressure at point A under 0.05gEI wave conditions.

**Fig 8:**
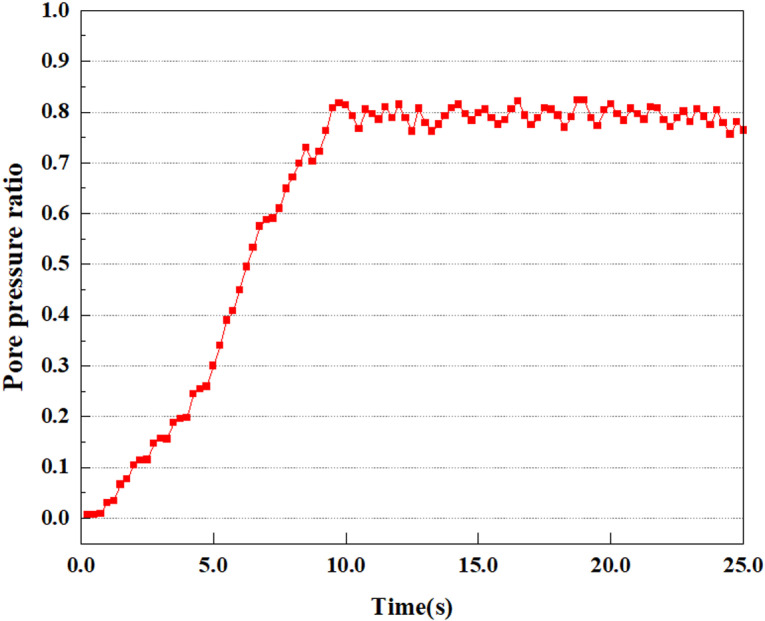
Variations in pore pressure ratio at point A under 0.05gEI wave conditions.

**Fig 9:**
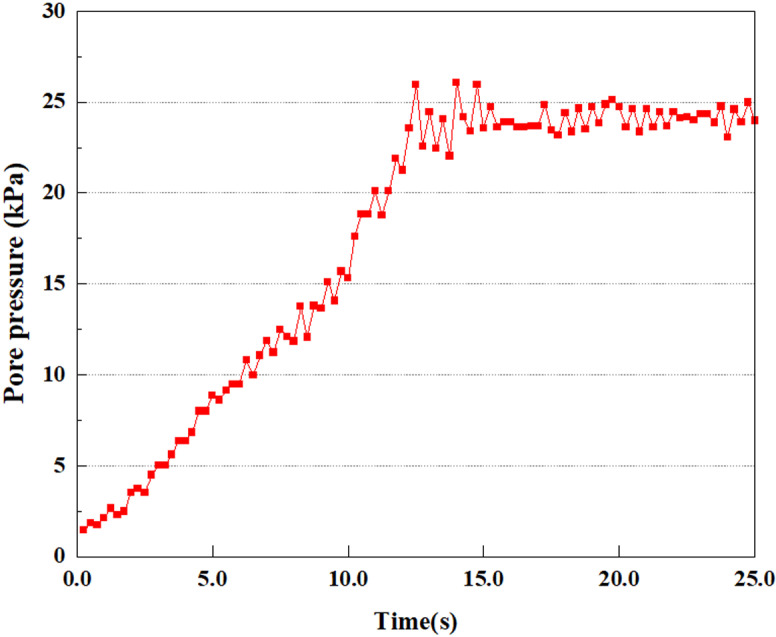
Variations in pore water pressure at point B under 0.05gEI wave conditions.

**Fig 10:**
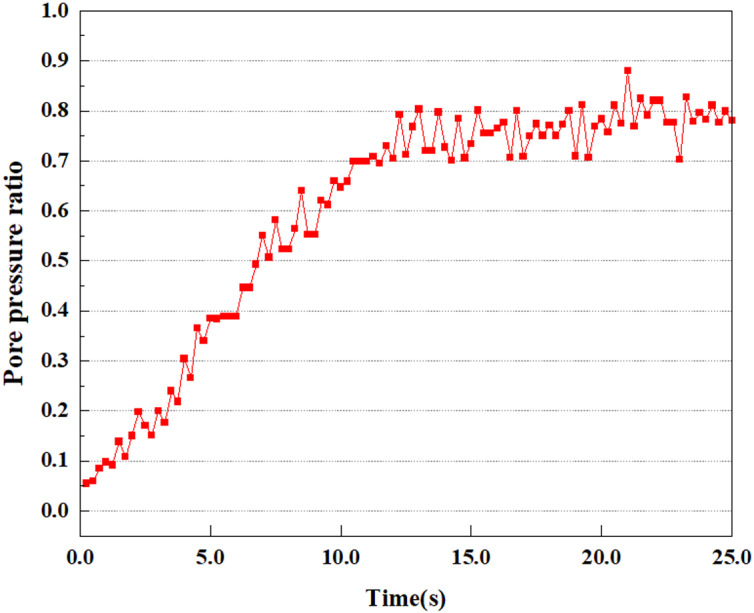
Variations in pore pressure ratio at point B under 0.05gEI wave conditions.

**Fig 11:**
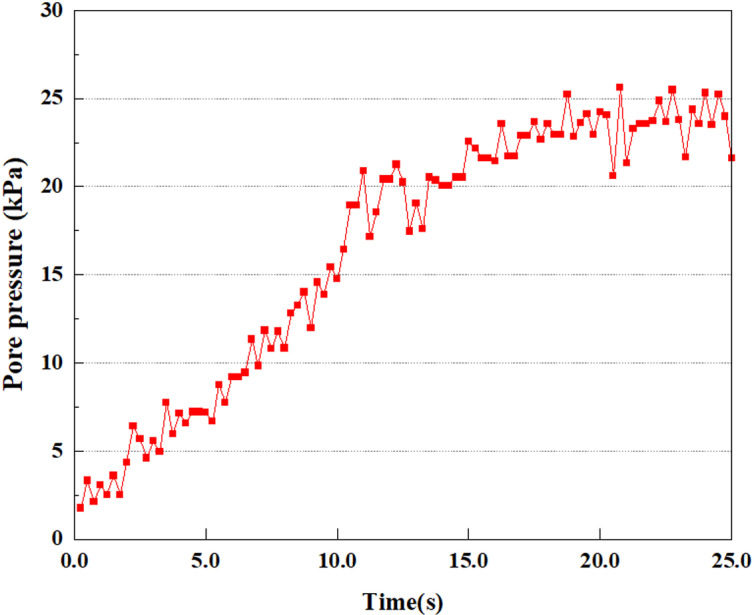
Variations in pore water pressure at point C under 0.05gEI wave conditions.

**Fig 12:**
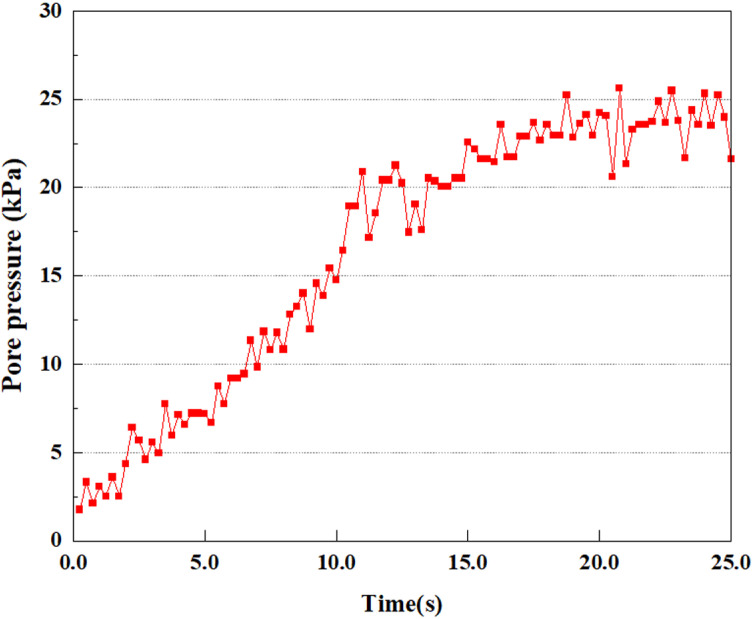
Variations in pore pressure ratio at point C under 0.05gEI wave conditions.

**Fig 13:**
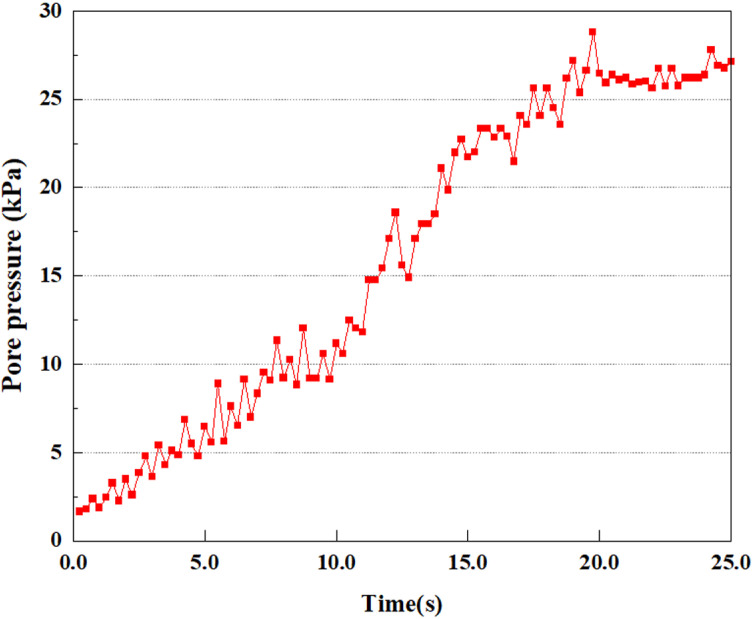
Variations in pore water pressure at point D under 0.05gEI wave conditions.

**Fig 14:**
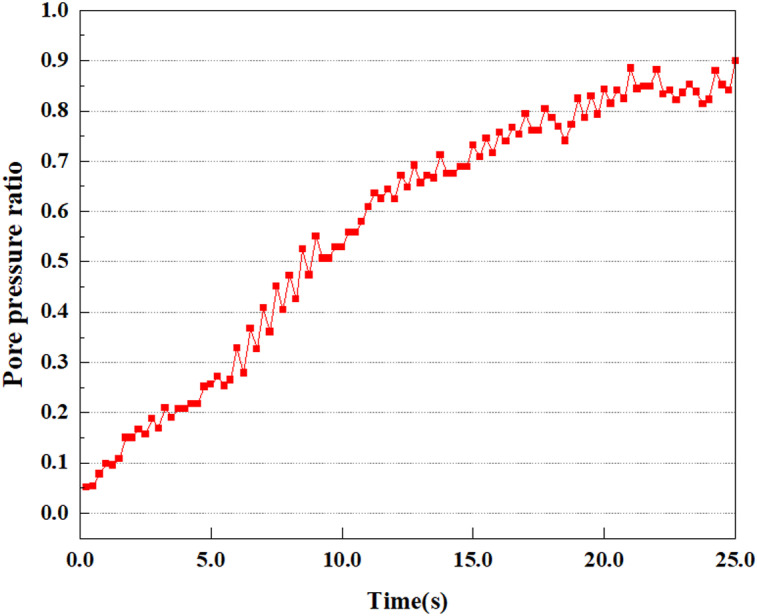
Variations in pore pressure ratio at point D under 0.05gEI wave conditions.

Set the dynamic load to 0.05gEI wave condition and apply it to each characteristic point, and extract the outcomes of pore pressure ratio along with soil pore water pressure for this condition. Figs 7–14 displays the variation in pore pressure ratio and pore water pressure under various seismic magnitudes for each characteristic point.

Pore pressure ratio is the ratio of pore water pressure to the effective stress in the formation, and is an important seismic indicator [37]. It is an indicator for the assessment of the soil’s liquefaction tendency under earthquake action, and the liquefaction tendency of soil can be reflected by the pore pressure ratio. In addition, the pore pressure ratio variation is also one of the focuses of research. Pore pressure ratio (PPR) is an essential index for assessing the deformation characteristics and stability of soil, reflecting the correlation between the effective stress fv and internal pore water pressure in the soil body. PPR is the ratio of the discrepancy between the soil’s initial and current effective stresses to the initial effective stress. The formula for calculating the pore pressure ratio is:


$$PPR=\frac{△P_{W}}{△P_{o}}=\frac{P_{o}-P^{B}}{P_{o}}$$ (2)

Among them, $△P_{W}$ is an increase in pore water overpressure; $P_{o}$ is the soil’s initial effective pressure; $P^{B}$ is the current effective stress.

The term “pore water pressure” describes the force of groundwater in rock or soil, which affects the pressure within the pores or between the particles directly. Set the dynamic load to 0.05gEI wave and apply it to each feature point. Under this condition, detailed extraction and statistical analysis were conducted on the soil’s PPR and pore water pressure to obtain comprehensive data. These data reflect the soil’s variations in pore water pressure under earthquake action. Figs 7-–14 contain the PPR variation at various magnitudes for each characteristic point under the 0.05gEI earthquake magnitude. It was observed that the PPR along with pore water pressure at feature point A increased rapidly from 0 to 10 seconds, stabilized at 0.8 and reached 23KPa at 12 seconds. The PPR together with pore water pressure of feature point B increase rapidly from 0 to 14 seconds, remain relatively stable at 15 seconds, but fluctuate significantly overall. The pore pressure ratio ranges from 0.75 to 0.9, and the pore water pressure reaches 24.5 KPa. For the feature point C, the pore water pressure and PPR increase from 0 to 18 seconds, and remain relatively stable after 19 seconds. The pore water pressure is between 20.5 Kpa and 25 KPa, and the PPR is essentially between 0.8 and 0.9. For the feature point D, the pore water pressure as well as PPR have been showing an increasing trend, with a slower growth within 0-7 seconds. Only a 0.3 rise in the pore pressure ratio and a mere 9 KPa rise in pore water pressure are observed. In 7 to 15 seconds, it grows quickly, and the PPR approaches 0.75. The pore pressure ratio begins to gradually raises after 15 seconds, reaching 0.9 and 26 KPa of pore water pressure after 25 seconds.

Overall, by applying a dynamic load of 0.05gEI wave, the soil’s variations in PPR and pore water pressure under different earthquake magnitudes were analyzed in depth. It was found that as the depth increases, the PPR and pore water pressure gradually rise, and the time to reach a stable value becomes later and later.

**Fig 15:**
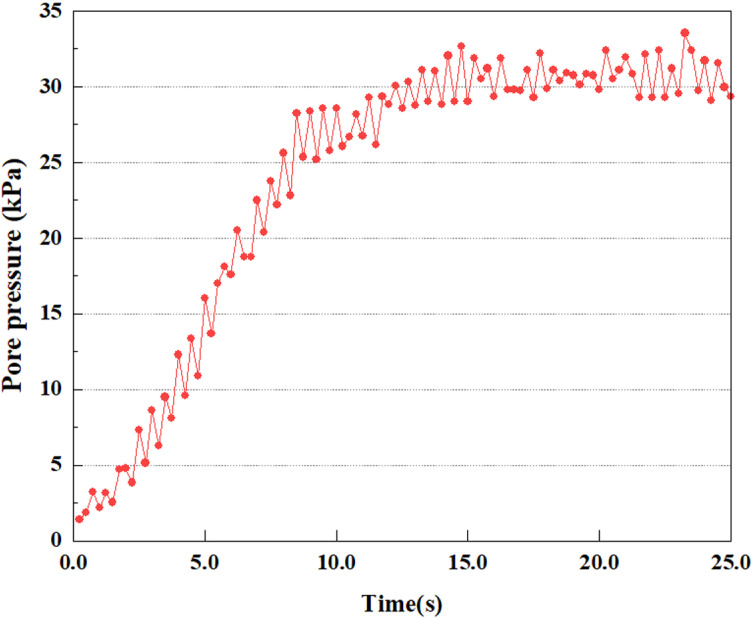
Variations in pore water pressure at point A under 0.25gEI wave conditions.

**Fig 16:**
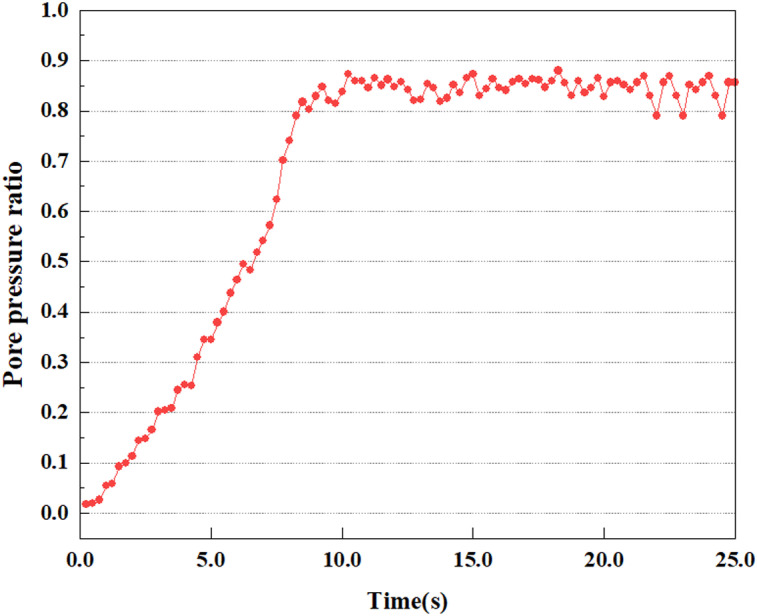
Variations in pore pressure ratio at point A under 0.25gEI wave conditions.

**Fig 17:**
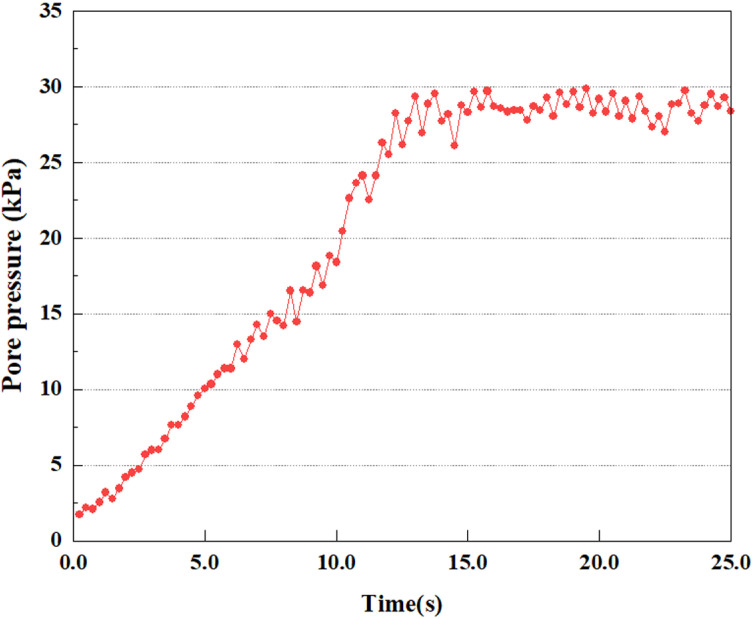
Variations in pore water pressure at point B under 0.25gEI wave conditions.

**Fig 18:**
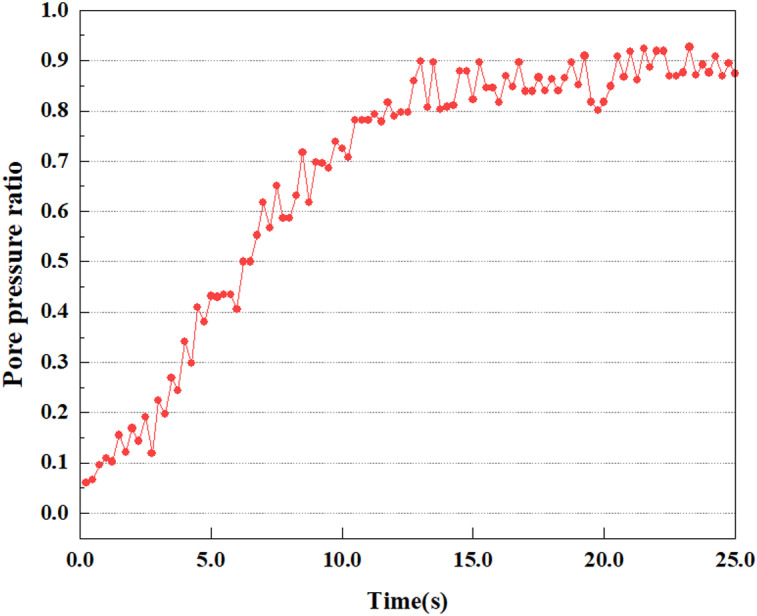
Variations in pore pressure ratio at point B under 0.25gEI wave conditions.

**Fig 19:**
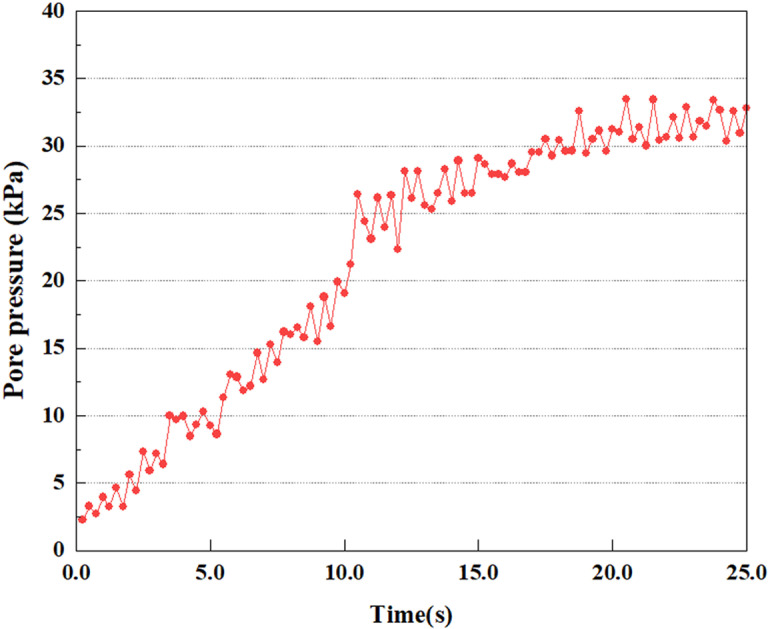
Variations in pore water pressure at point C under 0.25gEI wave conditions.

**Fig 20:**
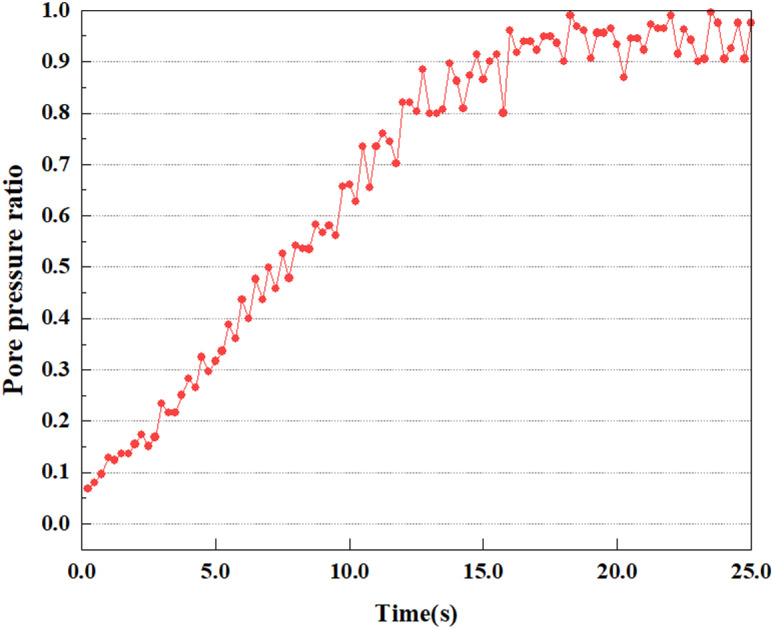
Variations in pore pressure ratio at point C under 0.25gEI wave conditions.

**Fig 21:**
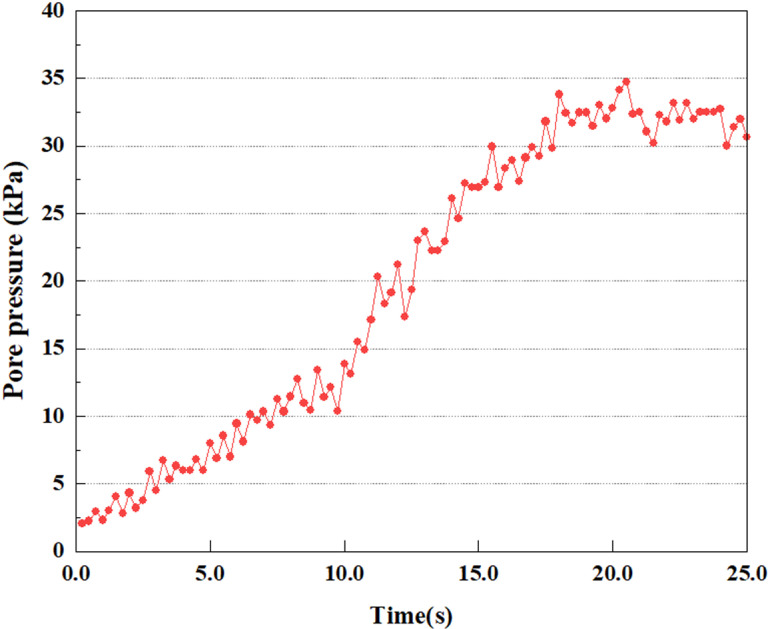
Variations in pore water pressure at point D under 0.25gEI wave conditions.

**Fig 22:**
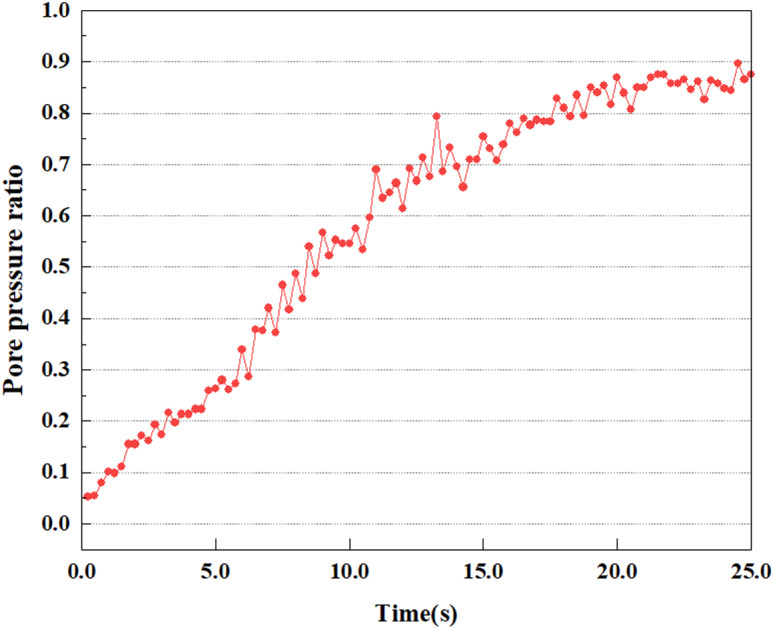
Variations in pore pressure ratio at point D under 0.25gEI wave conditions.

In the study, the dynamic load was set to the 0.25gEI wave condition and applied to various characteristic points to model the response of soil under the action of a large earthquake. Under this condition, the results of extracting soil PPR and pore water pressure were analyzed in detail. Figs 15–22 show the PPR and pore water pressure variations at various seismic magnitudes for each characteristic point. Through these graphs, the fluctuation of PPR and pore water pressure can be found under different seismic conditions.

Under the 0.25gEI wave condition, the PPR and pore water pressure variations of the soil may be more significant and complex, leading to significant deformation and compression inside the soil under larger seismic effects. By analyzing the PPR and pore water pressure data of each characteristic point, it was found that there were differences in the response of soil at different positions. The dynamic load was set to a 0.25gEI wave condition and applied to various characteristic points to study the PPR and pore water pressure variations of the soil under significant seismic action. Figs 15-–22 exhibit the PPR changes at diverse magnitudes for each characteristic point under a 0.25gEI earthquake magnitude. The Figure displays that the PPR at feature point A increases rapidly from 0 to 9 seconds, stabilizes at 9 seconds and reaches 0.84. The pore water pressure rapidly enhance in a range of 0-12 seconds, and after 12 seconds, it ranges from 27.5 Kpa to 31.5 Kpa. The PPR and pore water pressure of feature point B increase rapidly from 0 to 14 seconds, remain relatively stable at 14 seconds, but fluctuate significantly overall. The pore pressure ratio ranges from 0.85 to 0.94, and the pore water pressure ranges from 28Kpa to 30Kpa. The pore pressure ratio of feature point C increases from 0 to 17 seconds, and is relatively stable after 17 seconds, fluctuating within the range of 0.9 to 0.96. The pore water pressure is relatively stable after 20 seconds, ranging from 30KPa to 33Kpa. For the feature point D, the PPR and pore water pressure continue to increase from 0 to 21 seconds, until after 21 seconds, the pore water pressure stabilizes at around 33KPa and the PPR stabilizes at around 0.89. As shown in Figs 15–22, it is evident that the PPR and the trend of pore pressure variation are compatible.

**Fig 23:**
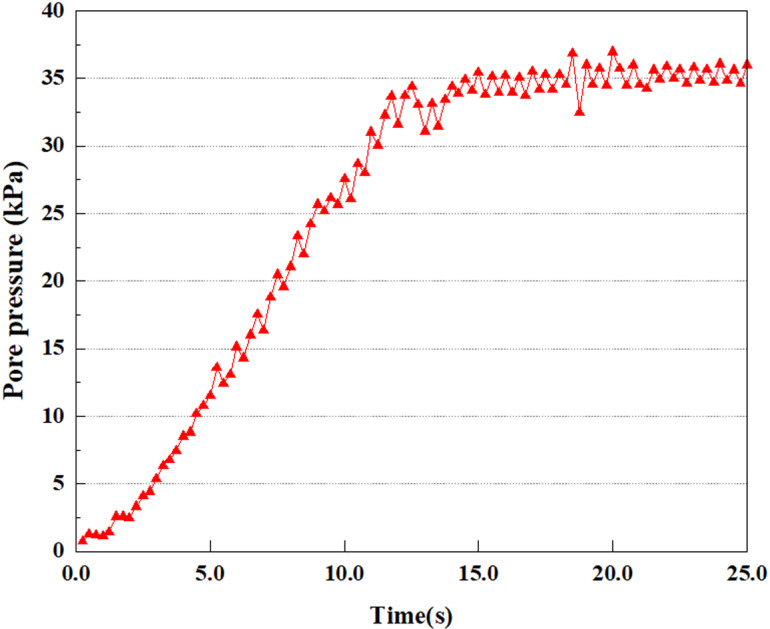
Changes in pore water pressure at point A under 0.50gEI wave conditions.

**Fig 24:**
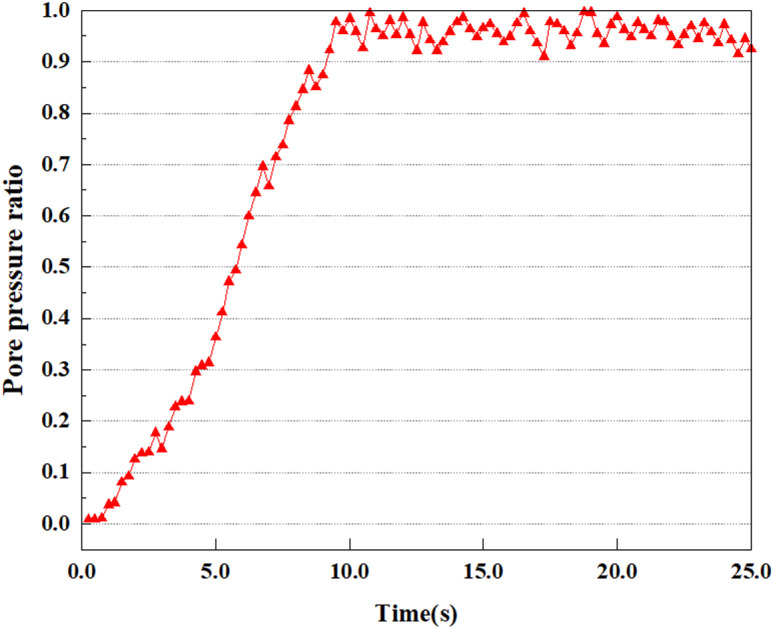
Changes in pore pressure ratio at point A under 0.50gEI wave conditions.

**Fig 25:**
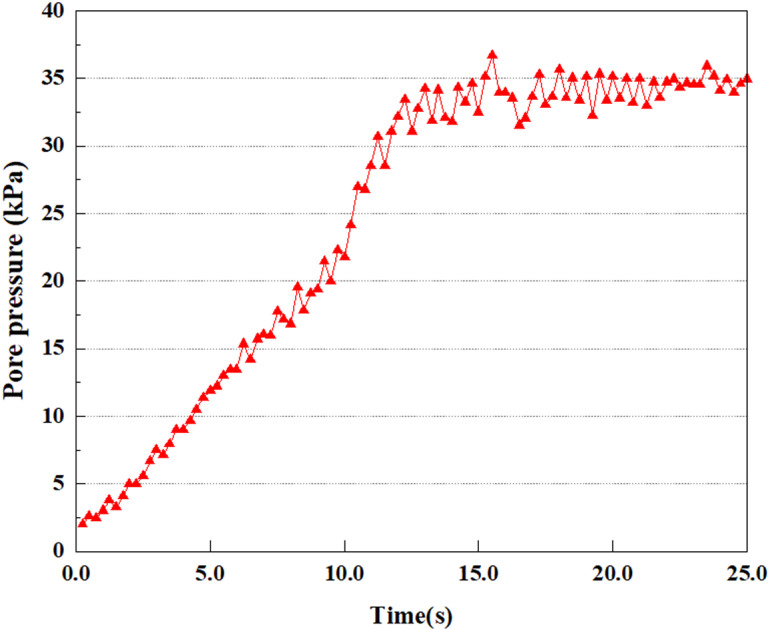
Changes in pore water pressure at point B under 0.50gEI wave conditions.

**Fig 26:**
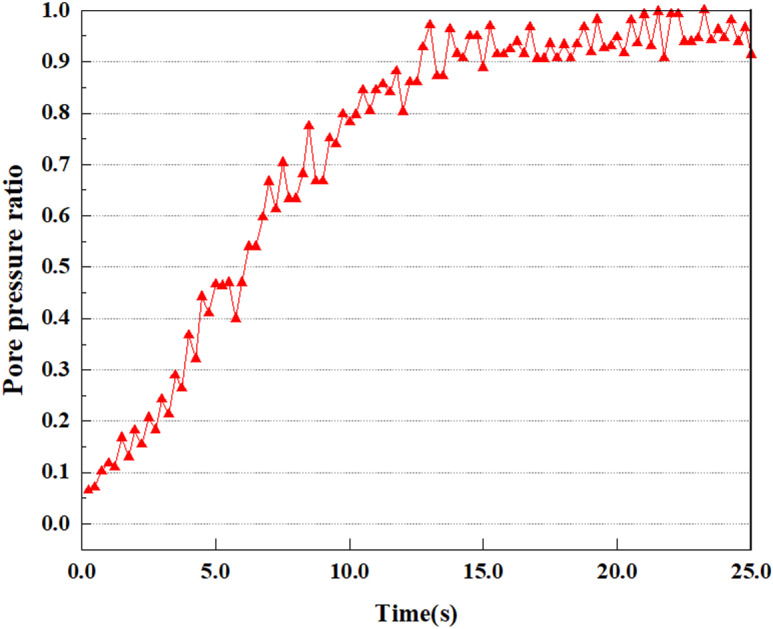
Changes in pore pressure ratio at point B under 0.50gEI wave conditions.

**Fig 27:**
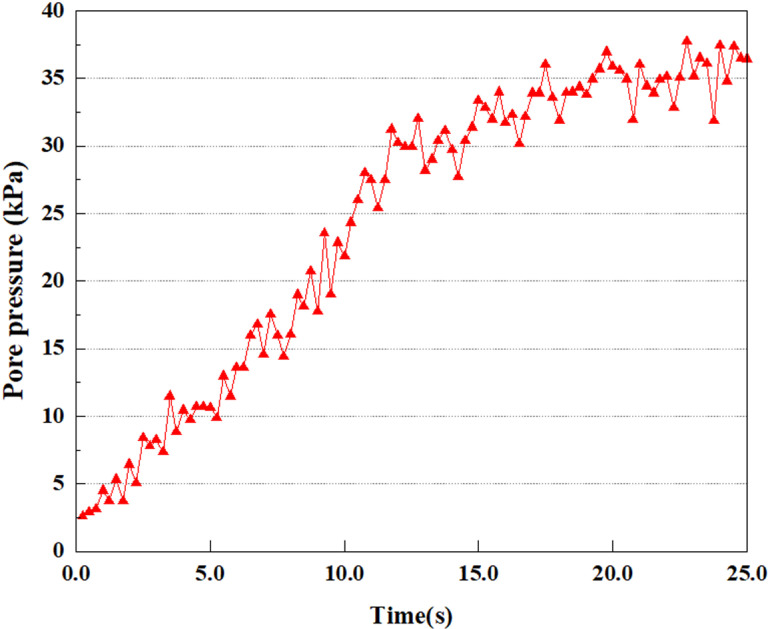
Changes in pore water pressure at point C under 0.50gEI wave conditions.

**Fig 28:**
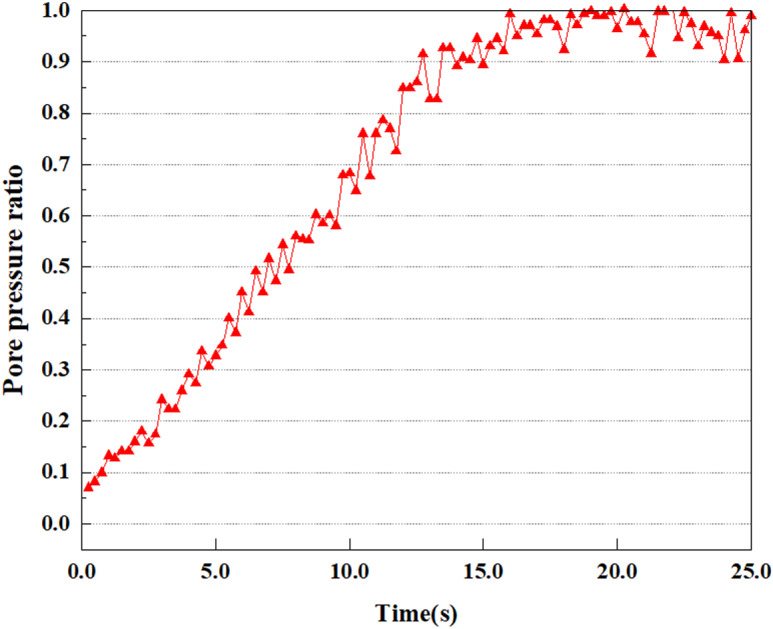
Changes in pore pressure ratio at point C under 0.50gEI wave conditions.

**Fig 29:**
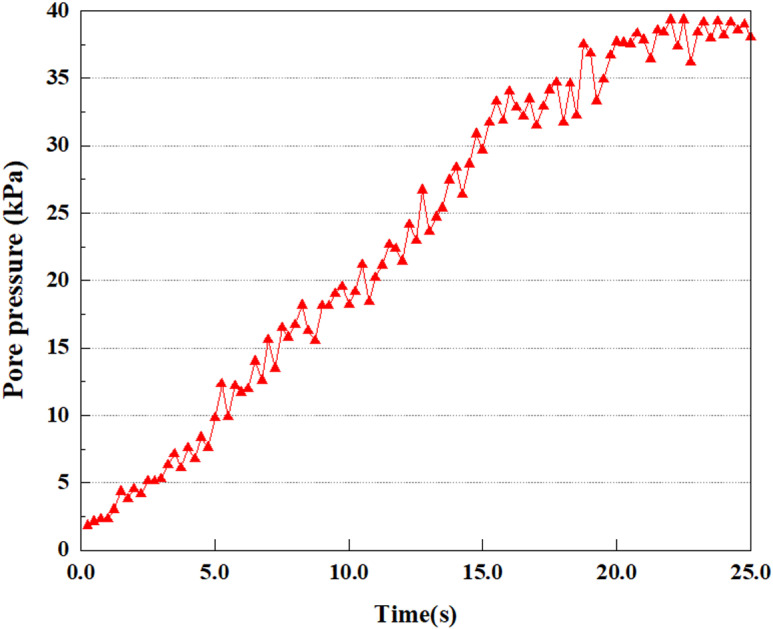
Changes in pore water pressure at point D under 0.50gEI wave conditions.

**Fig 30:**
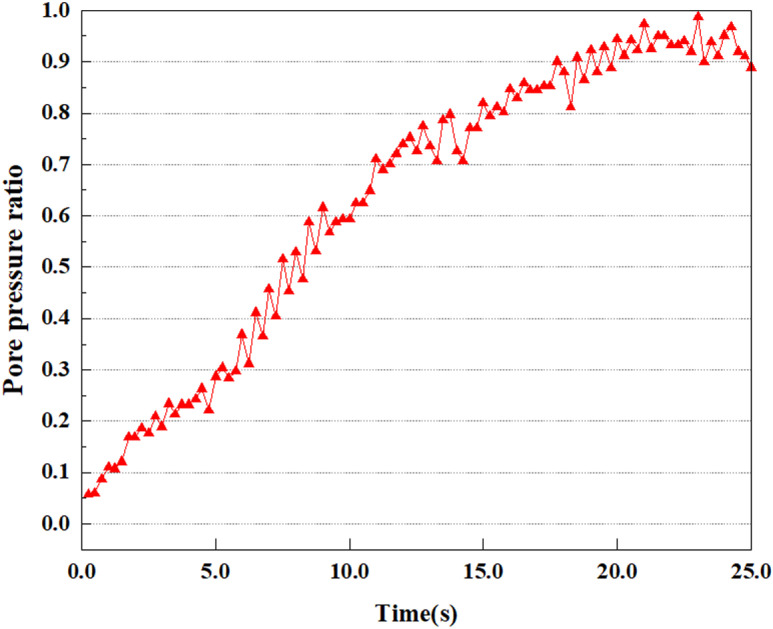
Changes in pore pressure ratio at point D under 0.50gEI wave conditions.

Simulation of soil response to extreme seismic actions under 0.50gEI wave loading conditions. The seismic action under these conditions will cause significant deformation and stress changes in the soil, making it crucial to study the soil’s PPR and pore water pressure. Figs 23–30 exhibit the PPR and pore water pressure variation at different seismic magnitudes for each characteristic point.

Under the condition of 0.50gEI wave, the soil will withstand greater seismic forces, causing a considerable rise in the soil’s internal pore pressure. This increase may cause significant deformation and compaction of the soil, thereby affecting the stability and engineering performance of the soil. It was observed that for the feature point A, the pore water pressure elevated from 0 to 12 seconds, and after 12 seconds, it was basically between 34Kpa and 35Kpa. The pore pressure ratio increased rapidly from 0 to 9 seconds, stabilized at 9 seconds, and reached 0.94. For the feature point B, the PPR and pore water pressure raise rapidly from 0 to 13 seconds, and remain relatively stable after 13 seconds. However, the overall fluctuation is significant, with pore water pressure ranging from 32.5 to 35 Kpa and PPR fluctuating between 0.89 and 0.97. For the feature point C, the PPR and pore water pressure elevate from 0 to 18 seconds, and remain relatively stable after 18 seconds. The pore water pressure fluctuates around 35 Kpa, and the pore pressure ratio fluctuates within the range of 0.9 to 0.98. For the feature point D, the PPR and pore water pressure have been revealing a raising trend, and the ultimate PPR and pore water pressure are is basically stable around 0.95 and at 39KPa, separately.

In summary, during the initial stage of vibration, the PPR and pore water pressure remain around 0, and after a period of time, they rapidly increase. The curve fluctuates up and down, and overall, the the PPR and pore water pressure gradually elevate. The the PPR and pore water pressure ultimately tend to stabilize. It could be observed that when the intensity of seismic vibration raises, the time when the PPR and pore water pressure begin to raise is earlier, and the time when stable the PPR and pore water pressure are ultimately achieved is also earlier. This is because as the intensity of seismic vibration increases, pore water pressure builds up more rapidly in sandy soils and the rise in pore water pressure is more pronounced, and the soil layer is more prone to deformation. In addition, the stabilized values of PPR and pore water pressure progressively grow as the intensity of seismic vibration rises. This is attributed to the fact that with the growth of seismic vibration intensity, the uplift value of pore water pressure becomes larger and closer to the soil’s effective stress. Earthquake magnitude is a key external factor affecting the stability of sandy soil. It was discovered that the PPR and pore water pressure of the upper soil layer rise earlier compared to the lower soil layer. This is attributed to the gradual transfer of surface vibrations to the lower soil during earthquake wave propagation. The upper soil is first affected by seismic waves, while the lower soil is only affected by earthquakes after a certain delay. Therefore, the upper soil is subjected to seismic forces earlier, and the PPR and pore water pressure start to grow earlier. Additionally, it may be caused by the damping action of the soil absorbing some of the seismic waves, resulting in the PPR and pore water pressure of the upper soil achieving stabilization earlier as compared to that of the lower soil. This is owing to the soil is composed of particles, and there is friction between the particles. When seismic waves pass through the soil, the friction between the particles will generate a certain amount of energy dissipation, thereby absorbing some of the seismic wave energy. Pore water in soil can also have a damping effect on seismic waves [38]. The flow, compression, and vibration of pore water consume energy, weakening the propagation of seismic waves.

### 3.2 Analysis of changes in pore pressure ratio and pore water pressure at the same characteristic point under different earthquake magnitudes

This article applies different magnitude loads to each feature point, and extracts the soil pore water pressure results for these conditions. Figs 31–38 illustrate the variation of PPR and pore water pressure at various magnitudes for each feature point.

In addition, this article summarizes the PPR and pore water pressure variations at the same characteristic point under different earthquake magnitudes in Table 2. With a constant pile position, the pore water pressure and PPR grow with elevating magnitude, as illustrated in the figure and table. The larger the magnitude, the longer it takes for the pore pressure and PPR to reach their peak. This may be because the greater the magnitude, the higher the dynamic load on the soil, and the pores between the soil are compressed. The particle structure tends to become compacted, and the pore water between the particles is compressed before it can be discharged, causing the pore water pressure to rise sharply, thereby augmenting the PPR. Meanwhile, we discovered that the PPR peaked later than the time when the pore water pressure peaked. This is explained by the fact that the PPR is the ratio of the pore water pressure at this time to the pore water pressure at the previous time, so it is inevitable that the pore pressure will peak first, and the PPR will follow the pore water pressure. Furthermore, it was noticed that the PPR and pore water pressure enhanced with the rise in pile depth. This may be due to the fact that with the growth in depth, the upper soil generates stress on the lower soil, and the transfer of stress can make it difficult to discharge pore water, hence enhancing the buildup of pore water pressure. Therefore, the lower soil is subjected to stress from the upper soil, causing an elevated pore water pressure.

**Table 2:** Variations in pore water pressure and PPR at different characteristic points under different earthquake magnitudes.

Feature points	Earthquake magnitude	Peak pore water pressure (KPa)	Time to reach Peak pore water pressure (s)	Peak pore pressure ratio	Time to reach peak pore pressure ratio (s)
A	0.05g	23.65	12	0.83	8.5
	0.25g	32.16	14	0.87	9
	0.50g	36.83	14.5	0.96	9.5
B	0.05g	26.31	12.5	0.84	10.5
	0.25g	29.86	12.5	0.91	11.5
	0.50g	37.13	14.5	0.96	14.5
C	0.05g	27.18	15	0.85	12.5
	0.25g	34.38	16.5	0.91	13.5
	0.50g	38.69	17	0.94	14
D	0.05g	28.03	17	0.86	17
	0.25g	38.64	18	0.92	18
	0.50g	39.73	19	0.96	19

### 3.3 Analysis of changes in pile acceleration at different feature points under the same magnitude

Set feature points 1, 2, 3, 4, and 5 at the bottom, middle, and top of the pile along the Z-axis direction. The distribution of feature points extracted from the pile acceleration [39] is shown in Fig 39. In the study, the dynamic load was set to the 0.05gEI wave condition and applied to various feature points to model the pile acceleration response under seismic action.

Figs 40–44 shows the changes in acceleration of each feature point under different magnitudes. It is possible to understand the trend of the acceleration of the pile body under the condition of 0.05gEI wave as a function of the magnitude, as well as the vibration situation at different characteristic points at different positions. From Figs 40–44, it can be noted that the response of peak acceleration at various locations of the pile body under the same earthquake intensity exhibits a distinctive trend with the characteristic point distribution. After analyzing the acceleration data at various locations on the pile, it was found that when the seismic intensity is the same, the value of the acceleration response of the pile body reduces as the depth of burial grows. The largest value of acceleration was observed at feature point 1, with a peak acceleration of 1.75m/s ². In contrast, the peak acceleration at the pile top is slightly greater than the acceleration at the pile bottom. This is because the position of the pile near the surface is more susceptible to seismic waves. The deeply buried part of the pile is less affected by seismic waves, while the shallower part is more significantly affected by earthquakes.

**Fig 31:**
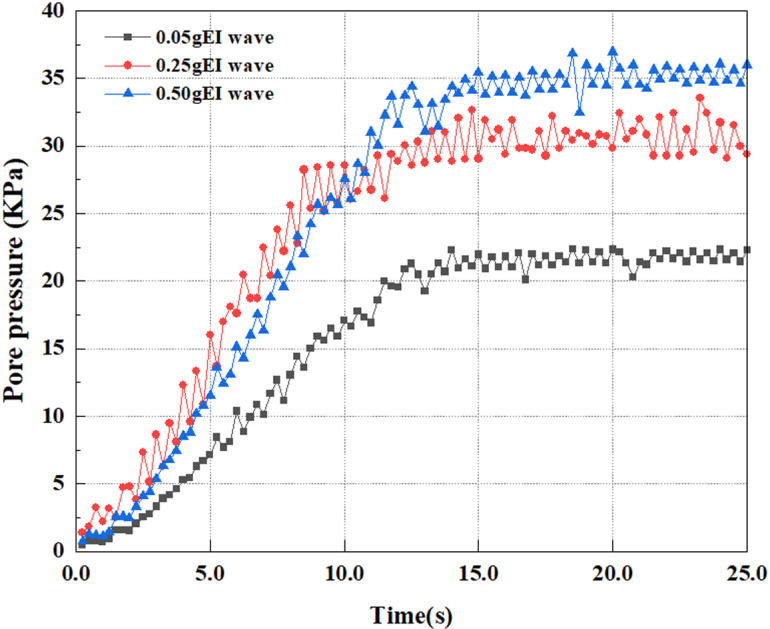
Pore water pressure variations at point A under different earthquake magnitudes.

**Fig 32:**
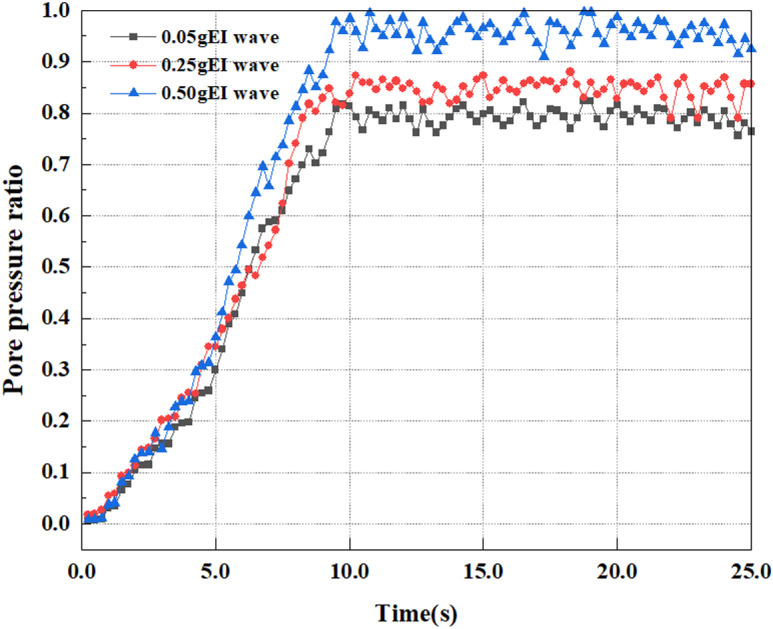
Pore pressure ratio variations at point A under different earthquake magnitudes.

**Fig 33:**
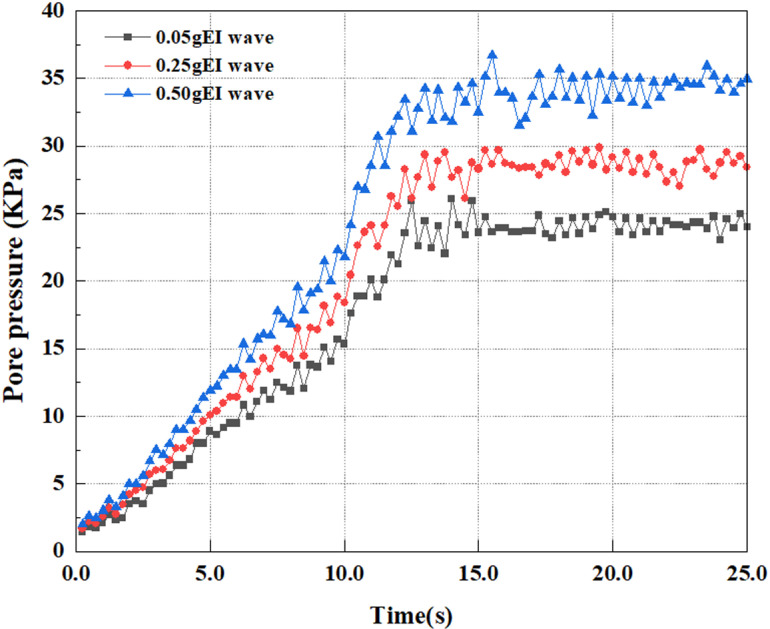
Pore water pressure variations at point B under different earthquake magnitudes.

**Fig 34:**
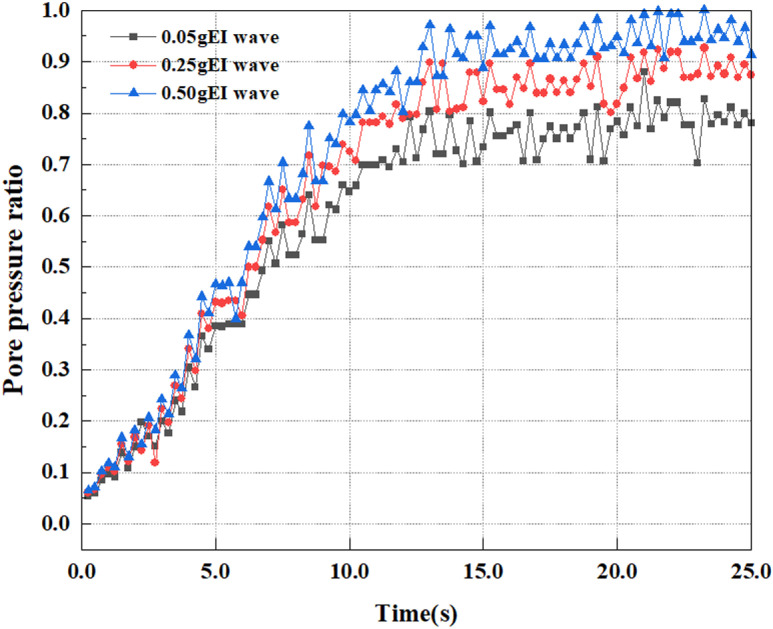
Pore pressure ratio variations at point B under different earthquake magnitudes.

**Fig 35:**
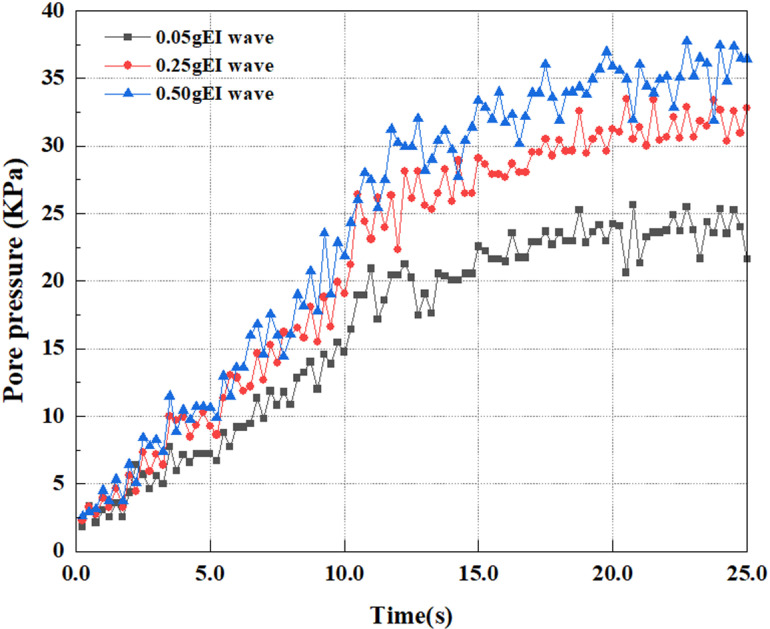
Pore water pressure variations at point C under different earthquake magnitudes.

**Fig 36:**
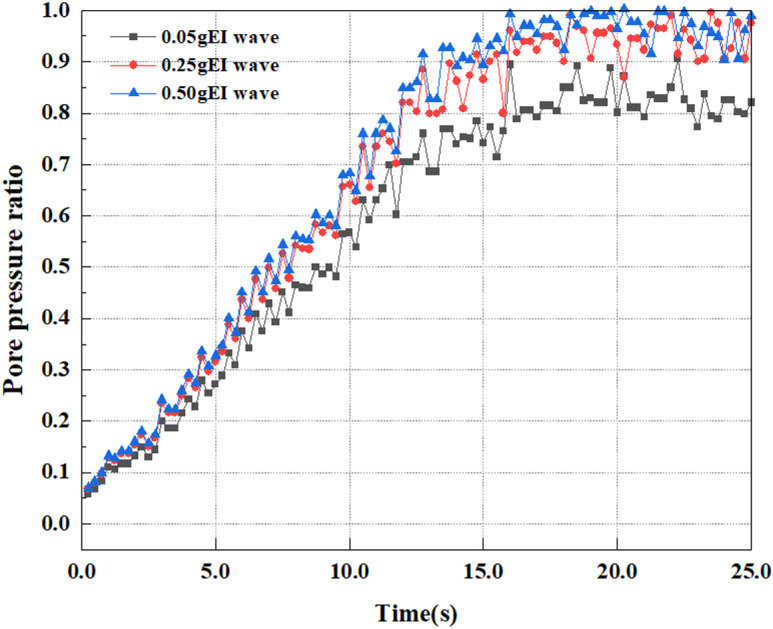
Pore pressure ratio variations at point C under different earthquake magnitudes.

**Fig 37:**
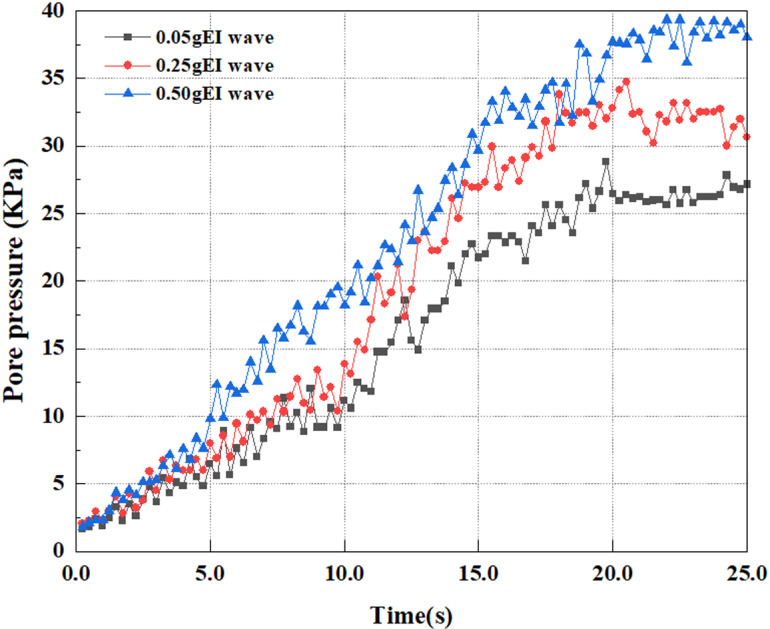
Pore water pressure variations at point D under different earthquake magnitudes.

**Fig 38:**
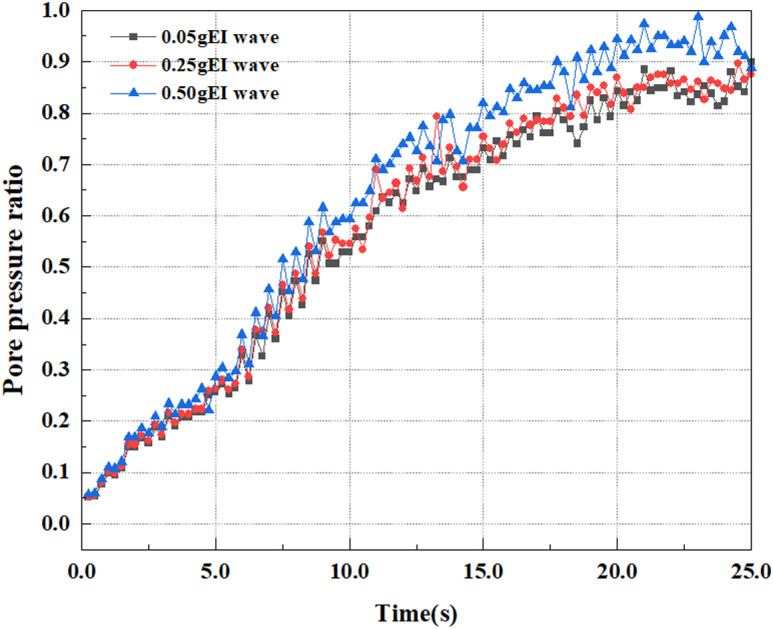
Pore pressure ratio variations at point D under different earthquake magnitudes.

**Fig 39:**
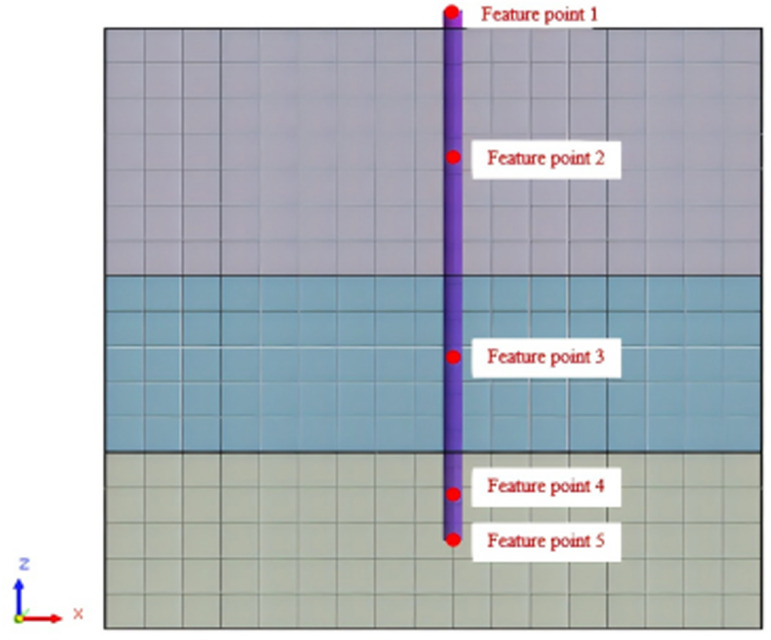
Distribution of feature points of pile acceleration.

**Fig 40:**
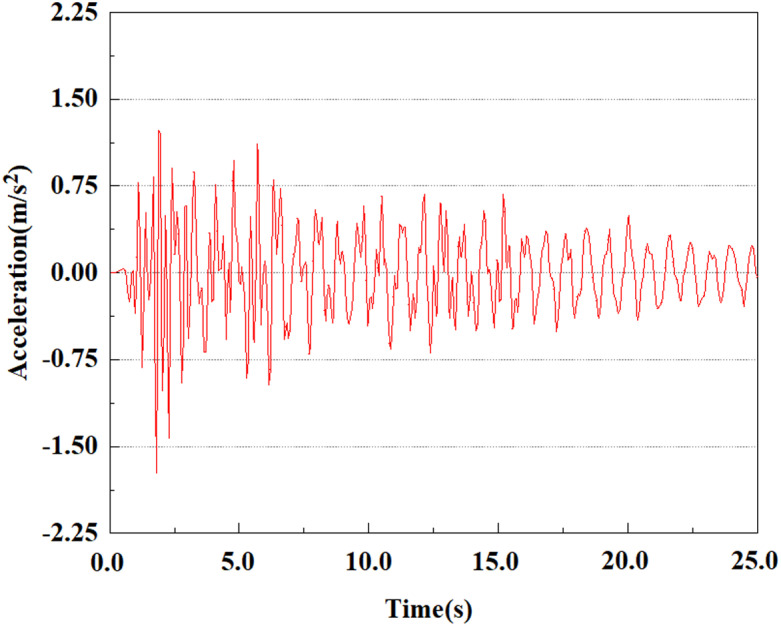
Changes in pile top acceleration at feature point 1 under 0.05gEI wave conditions.

**Fig 41:**
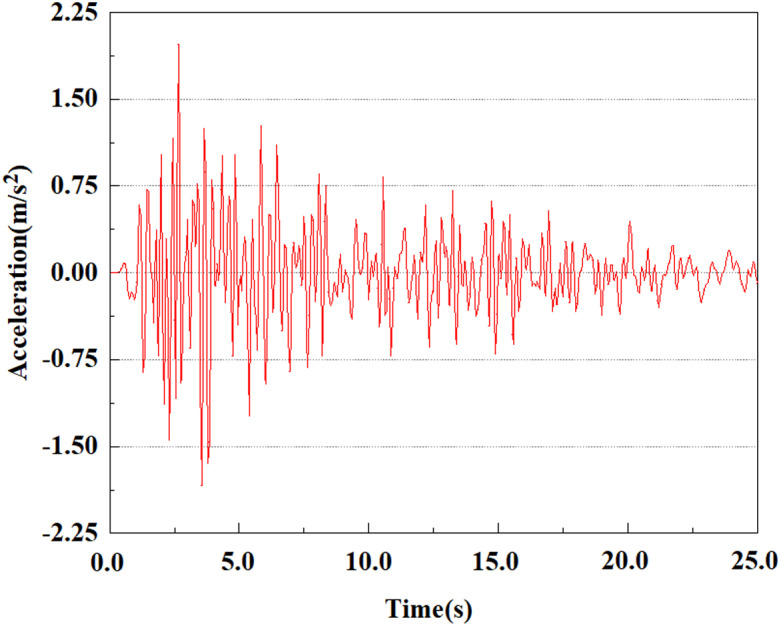
Changes in pile top acceleration at feature point 2 under 0.05gEI wave conditions.

**Fig 42:**
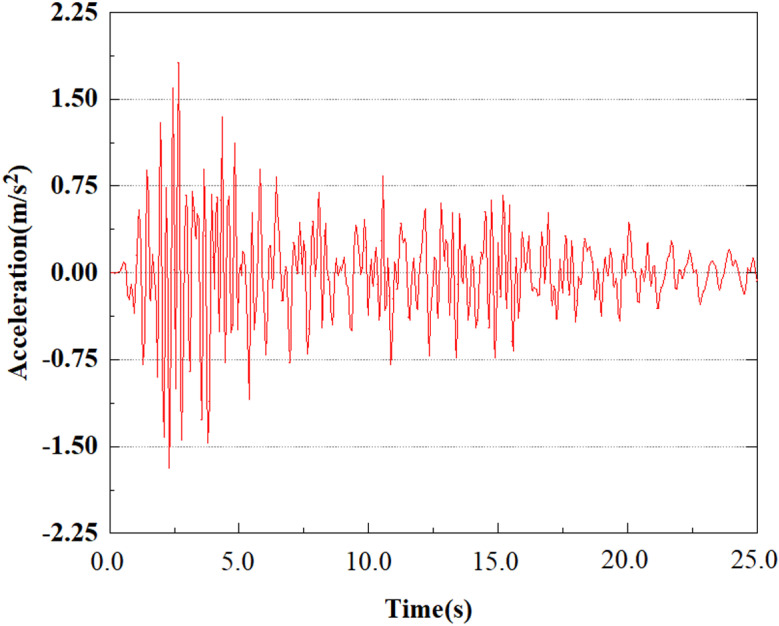
Changes in pile top acceleration at feature point 3 under 0.05gEI wave conditions.

**Fig 43:**
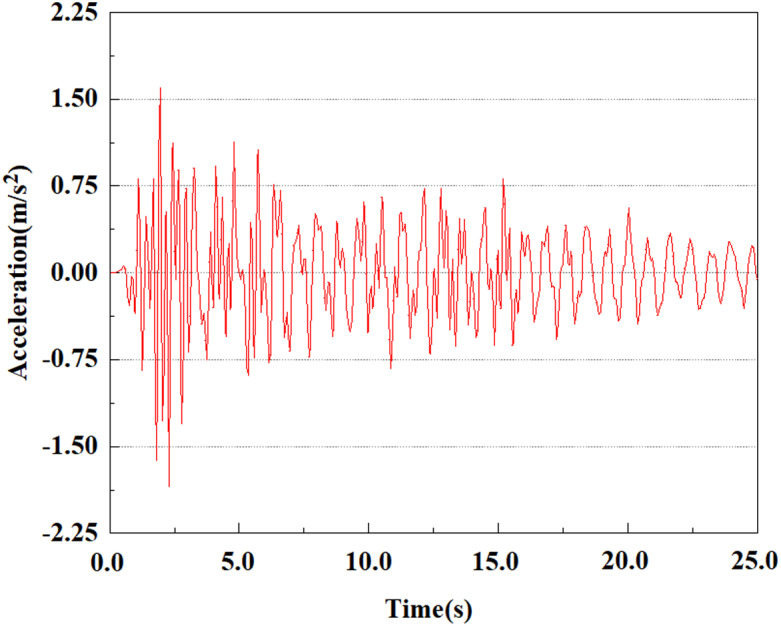
Changes in pile top acceleration at feature point 4 under 0.05gEI wave conditions.

**Fig 44:**
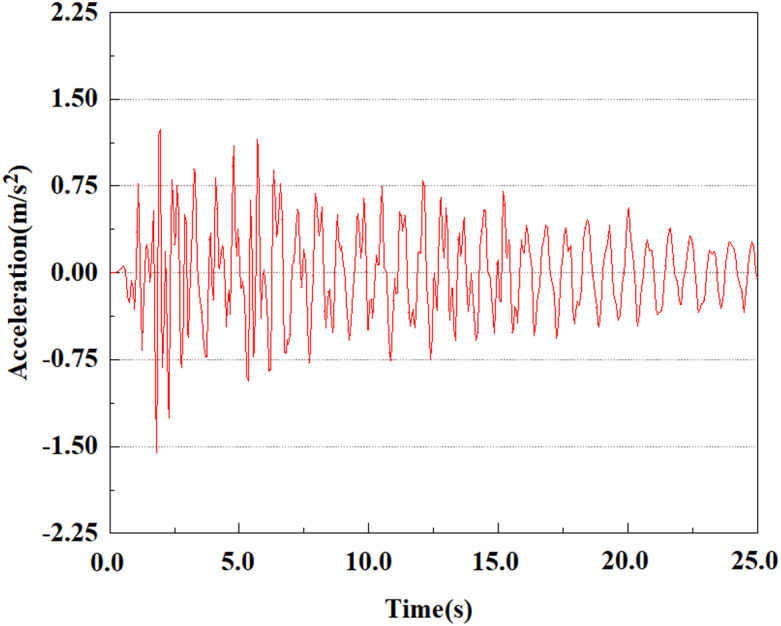
Changes in pile top acceleration at feature point 5 under 0.05gEI wave conditions.

**Fig 45:**
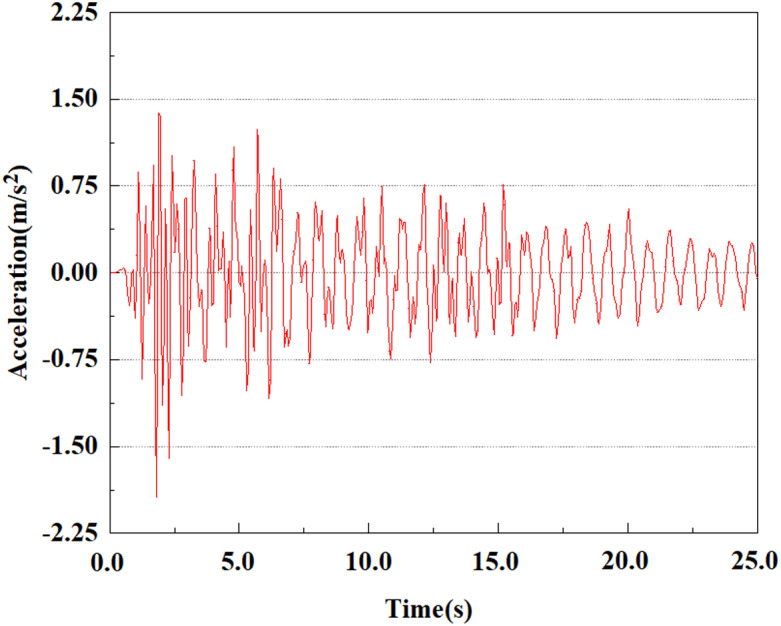
Changes in pile top acceleration at feature point 1 under 0.25gEI wave conditions.

**Fig 46:**
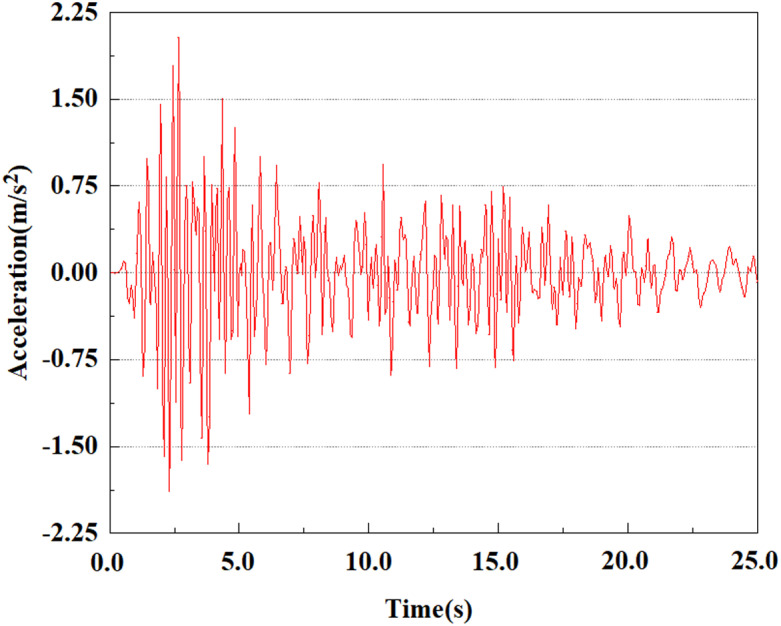
Changes in pile top acceleration at feature point 2 under 0.25gEI wave conditions.

**Fig 47:**
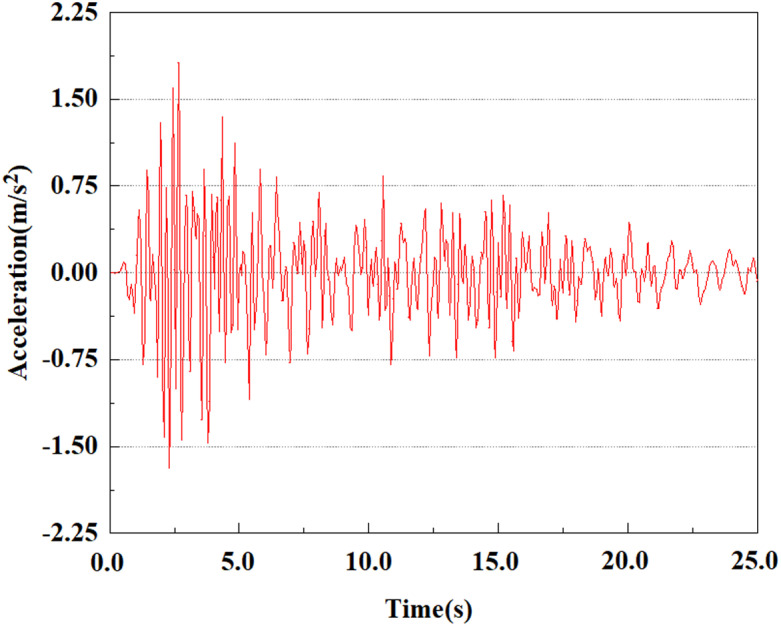
Changes in pile top acceleration at feature point 3 under 0.25gEI wave conditions.

**Fig 48:**
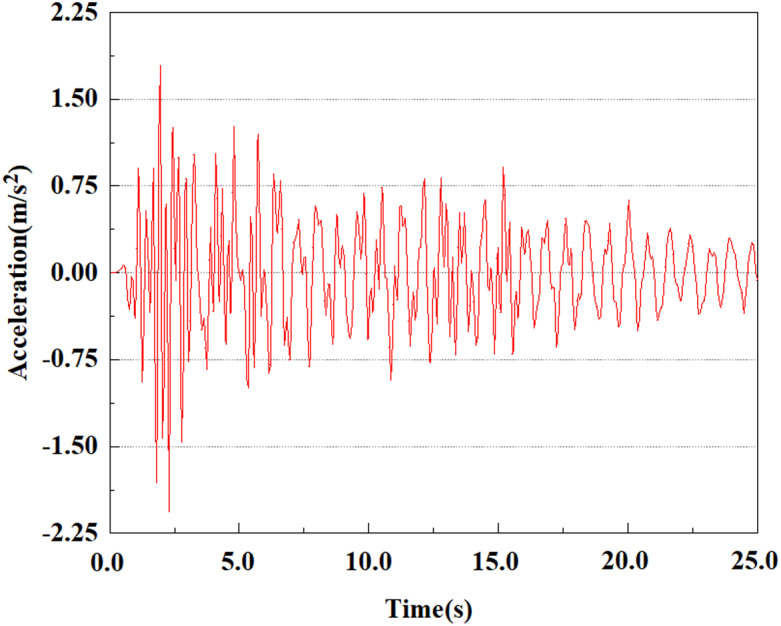
Changes in pile top acceleration at feature point 4 under 0.25gEI wave conditions.

**Fig 49:**
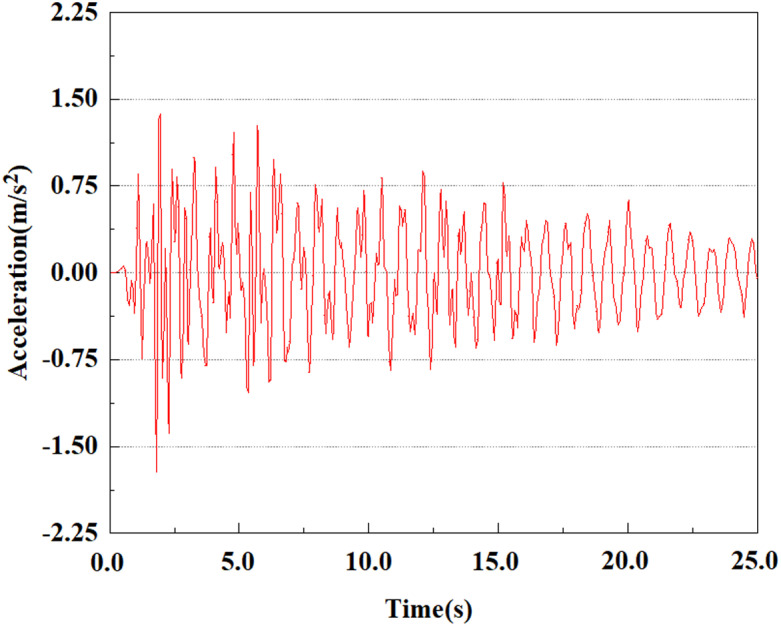
Changes in pile top acceleration at feature point 5 under 0.25gEI wave conditions.

Apply a dynamic load of 0.25gEI wave to each characteristic point to simulate seismic effects under different magnitudes. Under this condition, the accelerations at various feature points at different positions of the pile were extracted, and the acceleration changes of each feature point under different magnitudes were shown in Figs 45–49. It was found that under the 0.25gEI wave condition, acceleration response at various locations of the pile body showed a significant magnitude correlation. The peak acceleration of characteristic points increases correspondingly as the earthquake magnitude enhances, reflecting the effect of seismic intensity on the pile structure’s dynamic response. Under different magnitudes, the trend of acceleration changes at characteristic points also shows differences. The acceleration at different characteristic points shows a gentle change at different magnitudes, while at higher magnitudes it shows a more significant peak. This variation is linked to the dynamic properties of the structure and the response of the soil layer. It can be observed from Figs 45–49 that at the same intensity of seismicity, the response of the peak acceleration at different positions of the pile body varies with the distribution of characteristic points. This change reveals the influence of soil layer structure interaction. At characteristic point 1 in the sandy soil layer, it reached 2.04m/s ². The peak value of seismic waves, as well as the propagation of seismic waves in the sand layer with obvious pile-soil interaction, results in a significant acceleration response of the pile body. By examining the variations in acceleration at the pile top and bottom, it was noticed that the overall acceleration at the pile bottom was comparatively stable over time, and its peak value is smaller than other characteristic points. This is because the pile bottom is deeply buried underground and is buffered by the soil layer, which weakens the impact of seismic waves on the pile bottom.

**Fig 50:**
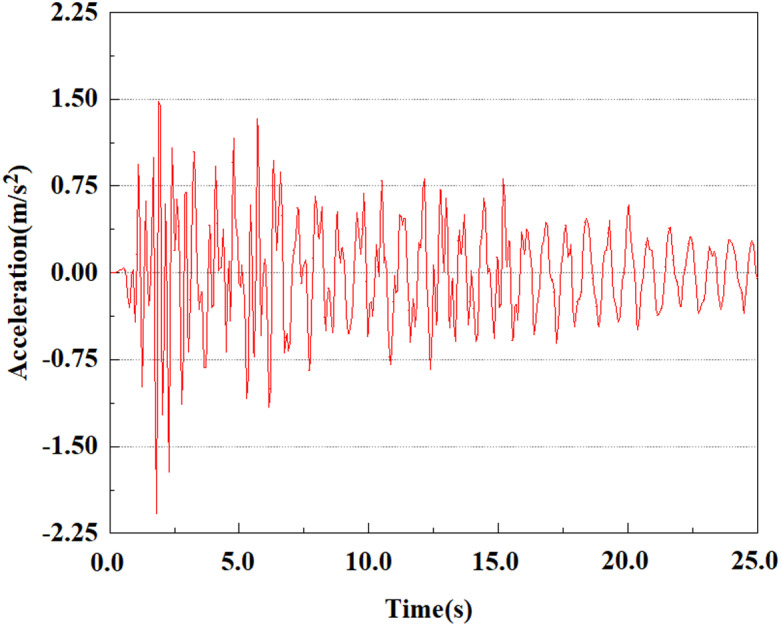
Changes in pile top acceleration at feature point 1 under 0.50gEI wave conditions.

**Fig 51:**
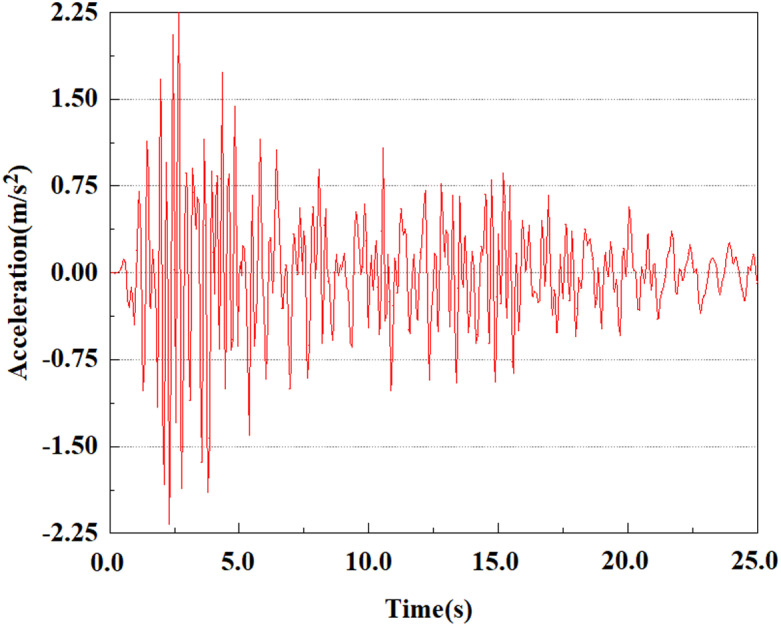
Changes in pile top acceleration at feature point 2 under 0.50gEI wave conditions.

**Fig 52:**
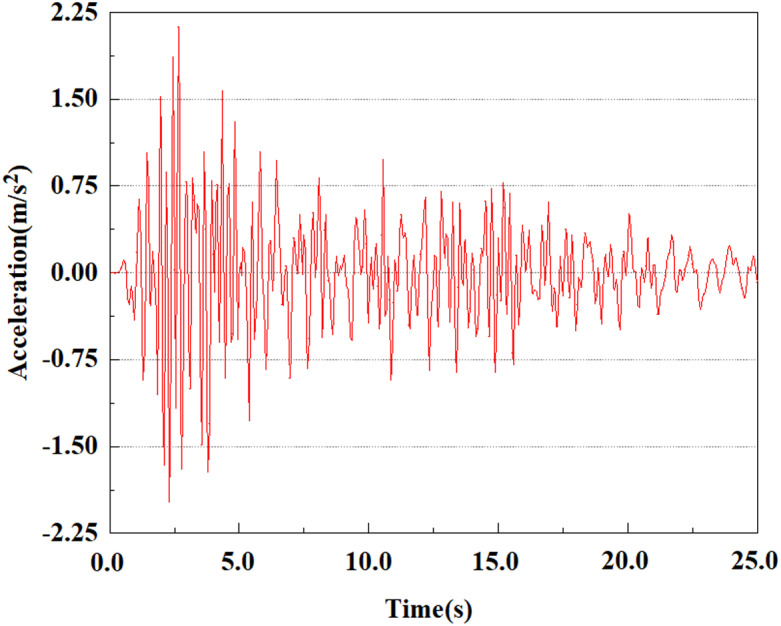
Changes in pile top acceleration at feature point 3 under 0.50gEI wave conditions.

**Fig 53:**
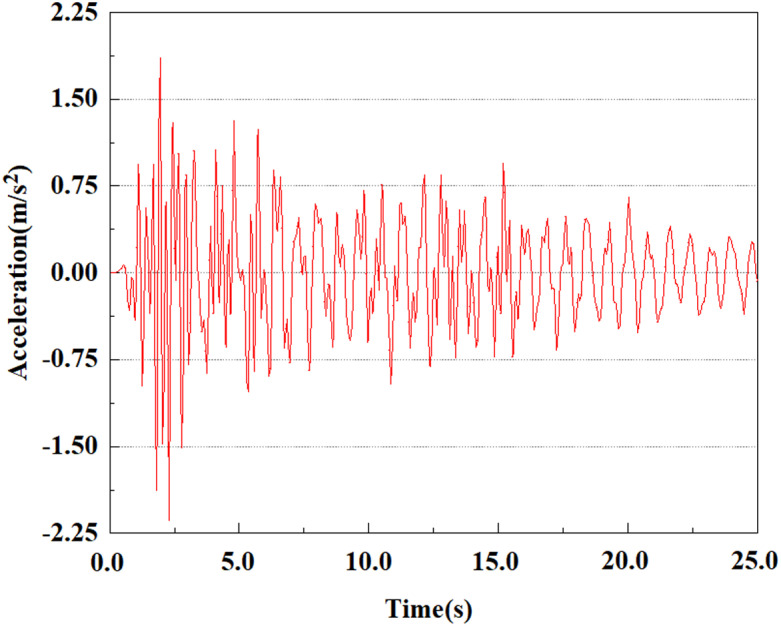
Changes in pile top acceleration at feature point 4 under 0.50gEI wave conditions.

**Fig 54:**
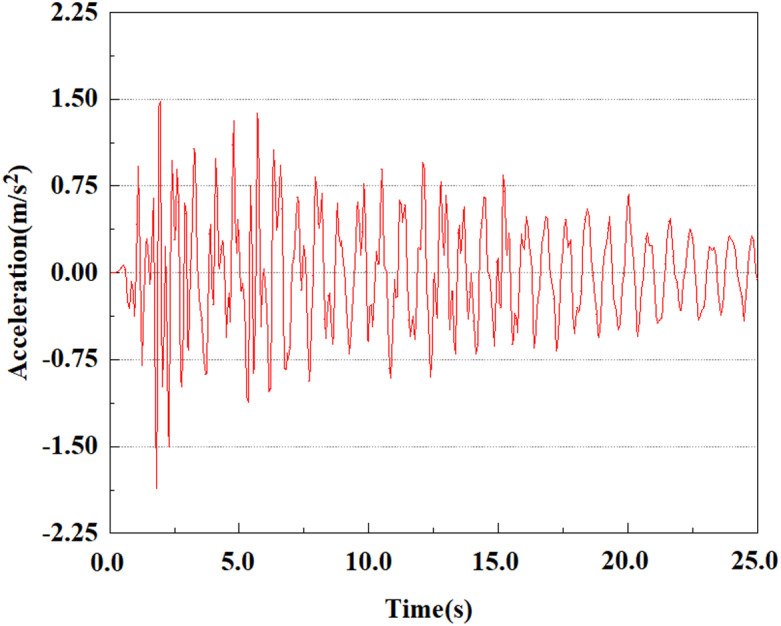
Changes in pile top acceleration at feature point 5 under 0.50gEI wave conditions.

Set the dynamic load to the 0.50gEI wave condition and apply it to each characteristic point. Figs 50–54 shows the acceleration changes of each feature point under the condition of 0.50gEI wave. These graphs can analyze the acceleration changes of feature points at different positions when the magnitude changes. As shown in Figs 50–54, at feature point 1, the maximum acceleration value is 2.21 m/s ², At feature point 5, the peak acceleration is 1.87m/s2. Under the same seismic intensity conditions, the pile body has remarkable variations in peak acceleration response at various locations, reflecting the pile’s dynamic response properties under seismic loading. With the depth of burial growing, the pile body exhibits decreasing trend of acceleration response value. This indicates that under the same seismic intensity, deeply buried piles are less affected by earthquakes than shallowly buried piles.

From the above analysis, it can be seen that under the same seismic magnitude, the rate of acceleration change at the pile top is greater than that at the pile bottom. This is because the soil absorbs seismic waves, resulting in a smaller load reaching the pile bottom than at the top. The damping action of the soil slows down the propagation of seismic waves, consequently lessening the acceleration response at the pile bottom because of the superior soil restrictions there. Because of the more direct seismic impact near the pile top, the peak acceleration there is somewhat higher than at the bottom. A single pile body’s acceleration rises steadily from the pile bottom to the top. At various locations of the pile body, the peak acceleration lags behind the peak acceleration of the input earthquake wave, and the acceleration response at the pile top is weaker than that at the pile bottom. In addition, we can see that the peak acceleration at feature point 5 shows a significant downward trend, while the peak acceleration at the other four feature points gradually increases. This phenomenon may be caused by the soil’s fixed constraint at the pile bottom, which results in a decrease in acceleration at the bottom of the pile.

### 3.4 Analysis of change of pile acceleration at the same feature point under different earthquake magnitudes

**Fig 55:**
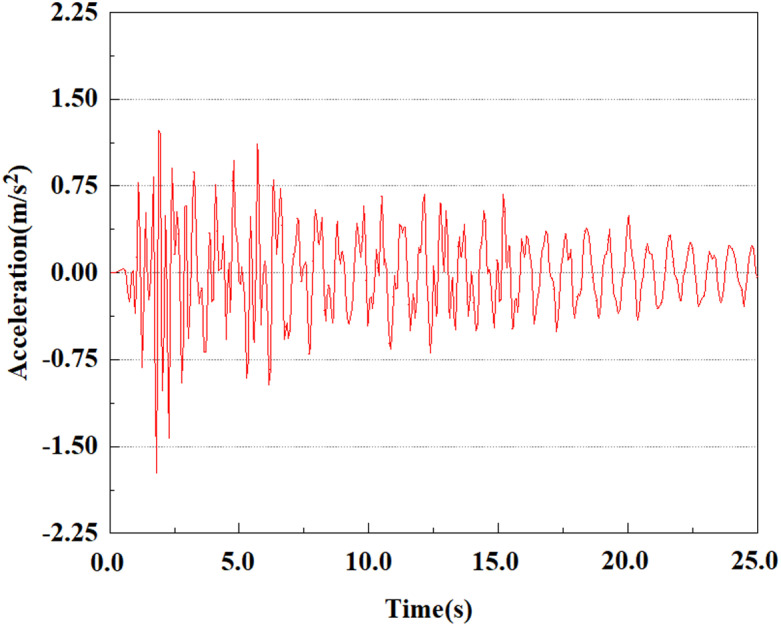
Changes in pile top acceleration under 0.05g EI wave magnitude at feature point 1.

**Fig 56:**
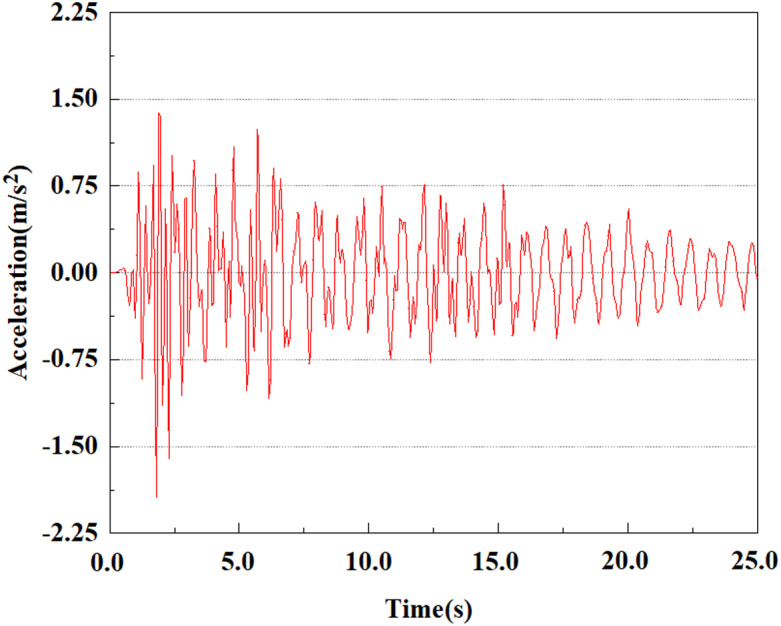
Changes in pile top acceleration under 0.25g EI wave magnitude at feature point 1.

**Fig 57:**
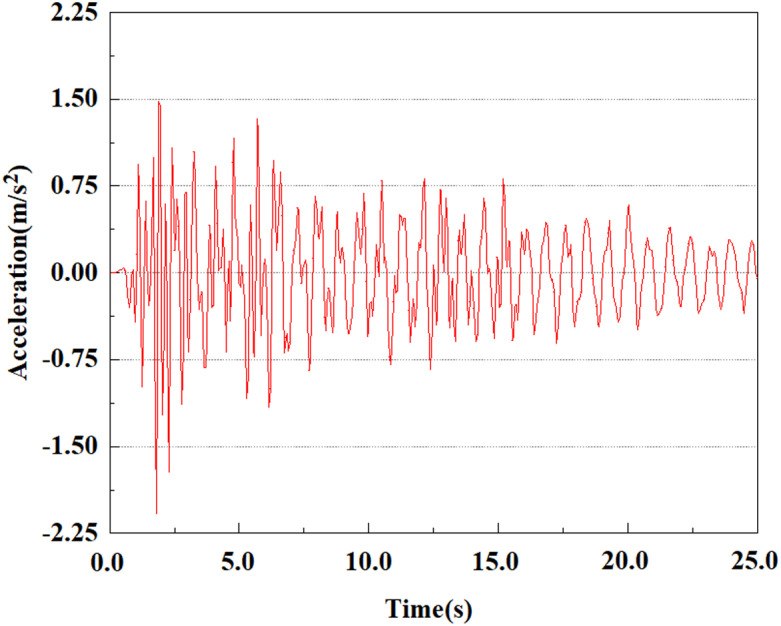
Changes in pile top acceleration under 0.50g EI wave magnitude at feature point 1.

From the Figs 55–57, it can be observed that the peak acceleration response varies with earthquake magnitude at various seismic intensities at the same pile position. At a magnitude of 0.05g, the peak acceleration at feature point 1 reached 1.75m/s ²; At a magnitude of 0.25g, the peak acceleration at feature point 1 reached 1.86m/s ²; At a magnitude of 0.5g, the peak acceleration at feature point 1 reached 2.21m/s ². This suggests that the pile body’s peak acceleration grows in tandem with the earthquake’s amplitude.

**Fig 58:**
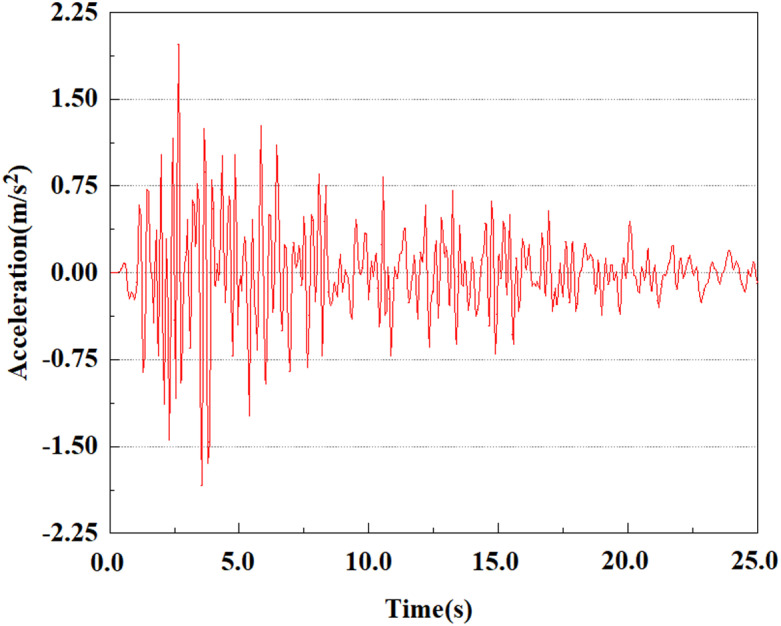
Changes in pile top acceleration under 0.05g EI wave magnitude at feature point 2.

**Fig 59:**
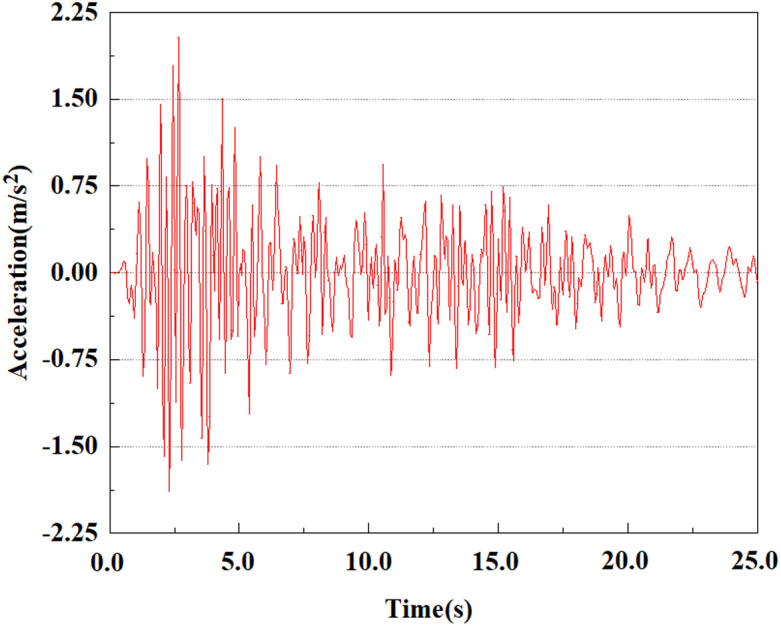
Changes in pile top acceleration under 0.25g EI wave magnitude at feature point 2.

**Fig 60:**
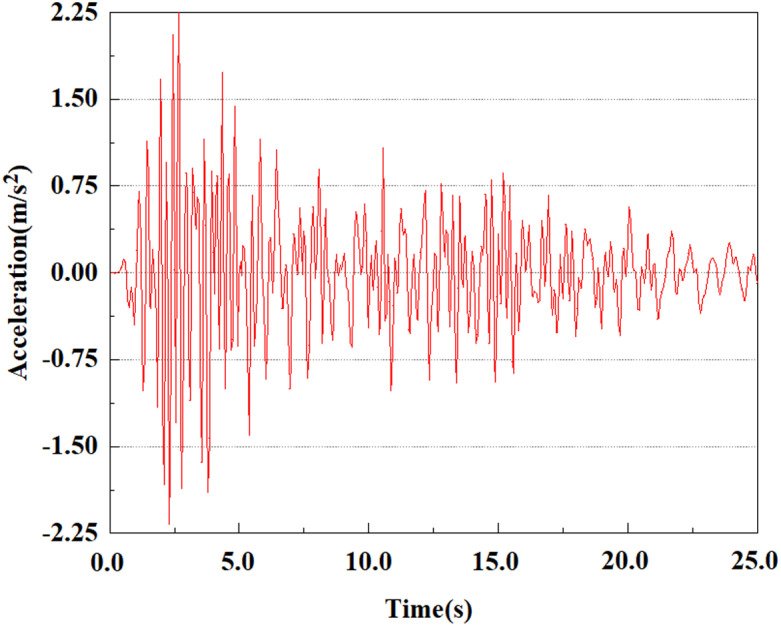
Changes in pile top acceleration under 0.50g EI wave magnitude at feature point 2.

From Figs 58–60, it is evident that the peak acceleration response varies with earthquake magnitude at various seismic intensities at the same pile position. At a magnitude of 0.05g, the peak acceleration at feature point 2 reached 1.81m/s ²; At a magnitude of 0.25g, the peak acceleration at feature point 2 reached 1.96m/s ²; At a magnitude of 0.5g, the peak acceleration at feature point 2 reached 2.41m/s ².

**Fig 61:**
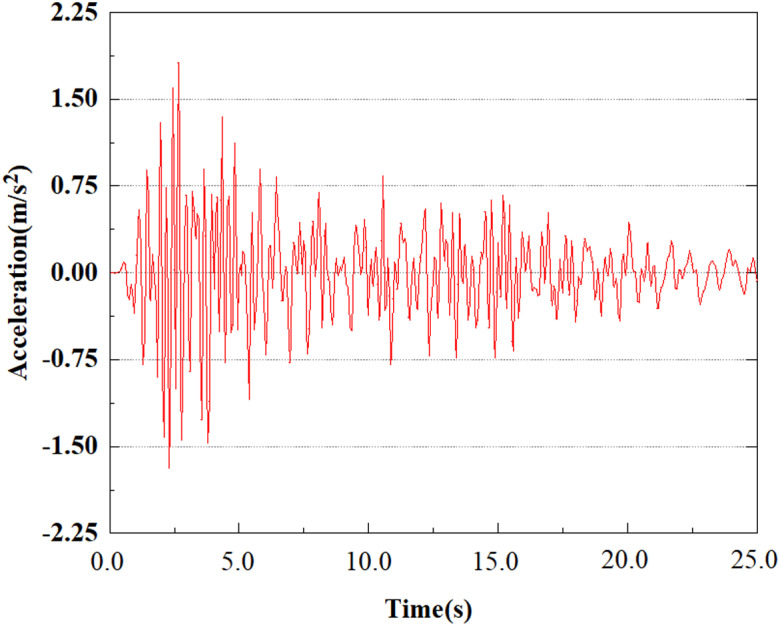
Changes in pile top acceleration under 0.05g EI wave magnitude at feature point 3.

**Fig 62:**
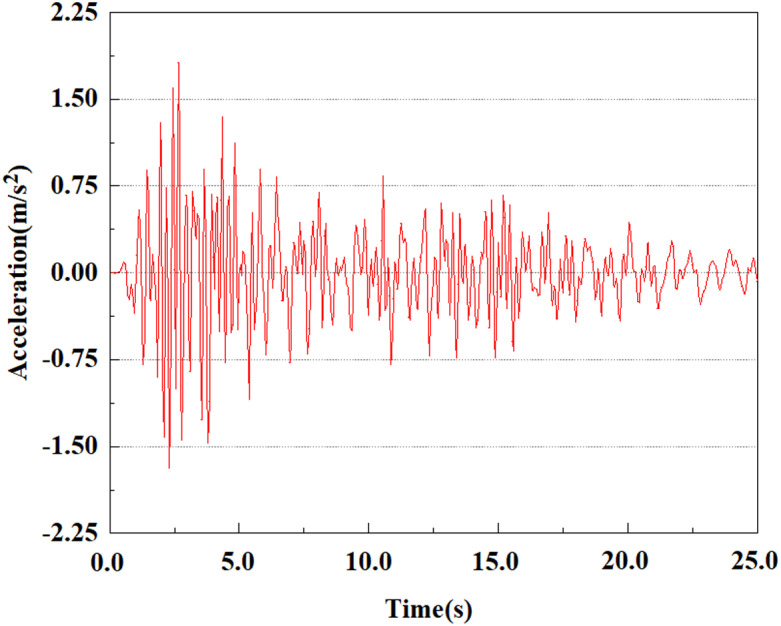
Changes in pile top acceleration under 0.25g EI wave magnitude at feature point 3.

**Fig 63:**
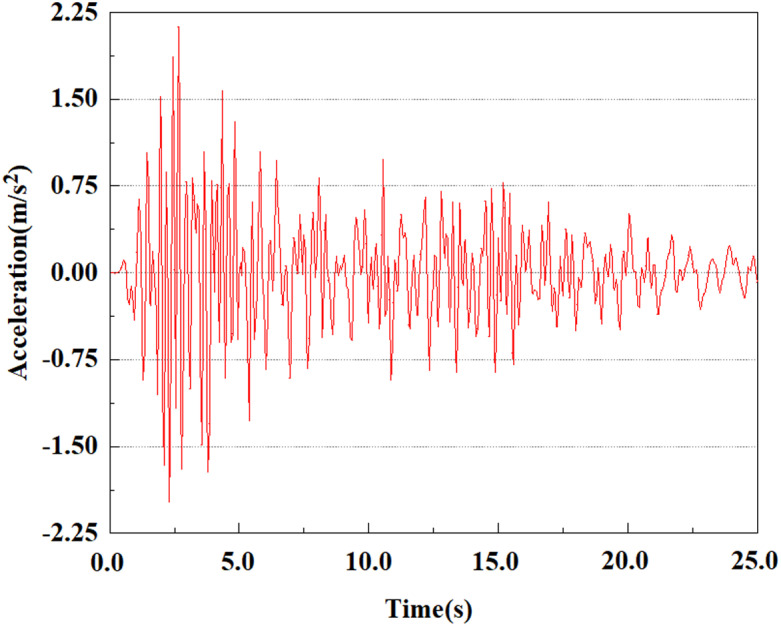
Changes in pile top acceleration under 0.50g EI wave magnitude at feature point 3.

From Figs 61–63, it is obvious that the peak acceleration response changes with earthquake magnitude at various seismic intensities at the same pile position. At a magnitude of 0.05g, the peak acceleration at feature point 3 reached 1.85m/s ²; At a magnitude of 0.25g, the peak acceleration at feature point 3 reached 2.16m/s ²; At a magnitude of 0.5g, the peak acceleration at feature point 3 reached 2.25m/s ².

**Fig 64:**
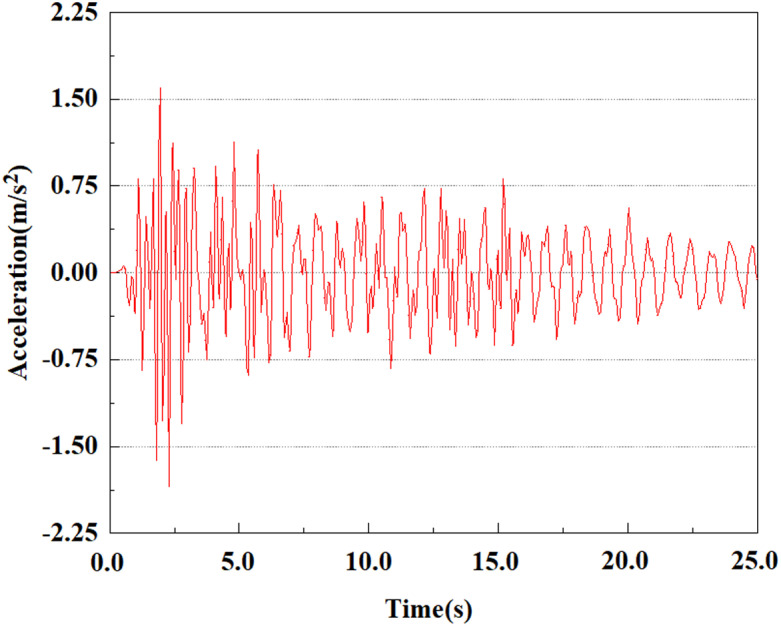
Changes in pile top acceleration under 0.05g EI wave magnitude at feature point 4.

**Fig 65:**
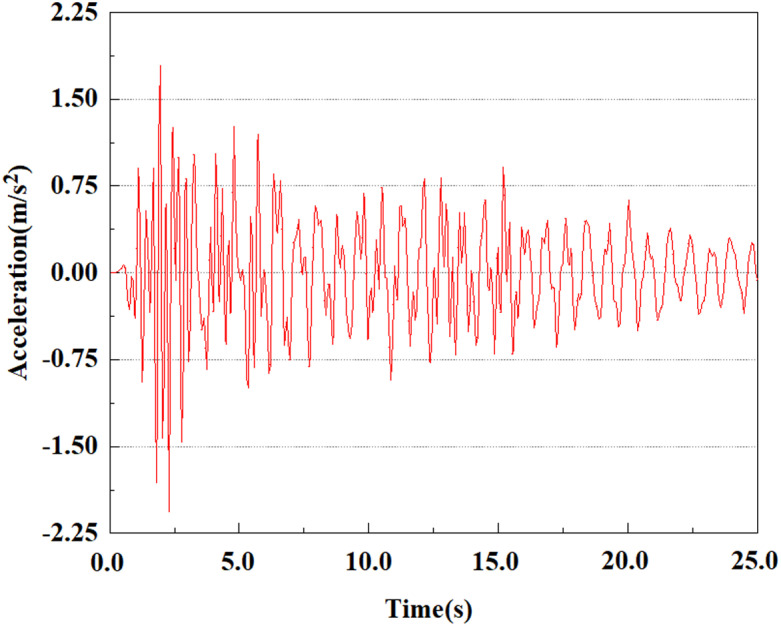
Changes in pile top acceleration under 0.25g EI wave magnitude at feature point 4.

**Fig 66:**
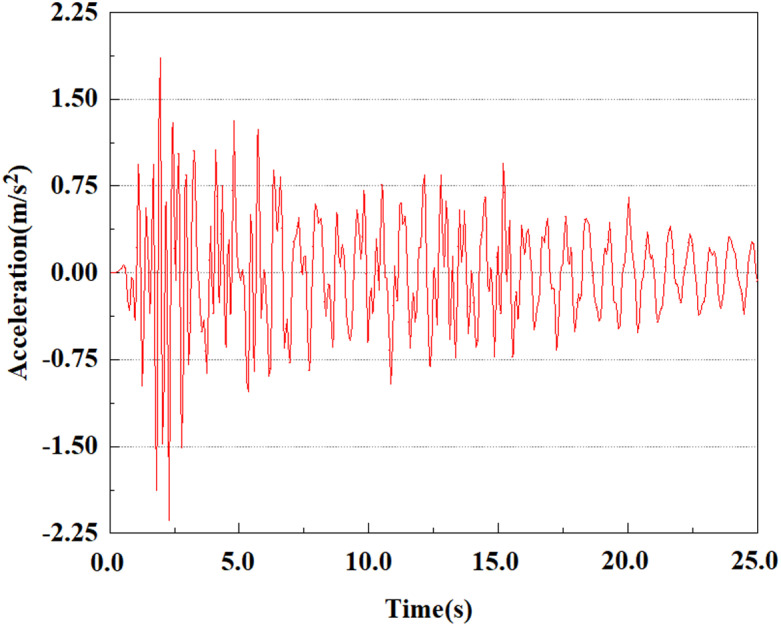
Changes in pile top acceleration under 0.50g EI wave magnitude at feature point 4.

From Figs 64–66, it is evident that the peak acceleration response varies with earthquake magnitude at various seismic intensities at the same pile position. At a magnitude of 0.05g, the peak acceleration at feature point 4 reached 1.86m/s ²; At a magnitude of 0.25g, the peak acceleration at feature point 4 reached 2.10m/s ²; At a magnitude of 0.5g, the peak acceleration at feature point 4 reached 2.21m/s ².

**Fig 67:**
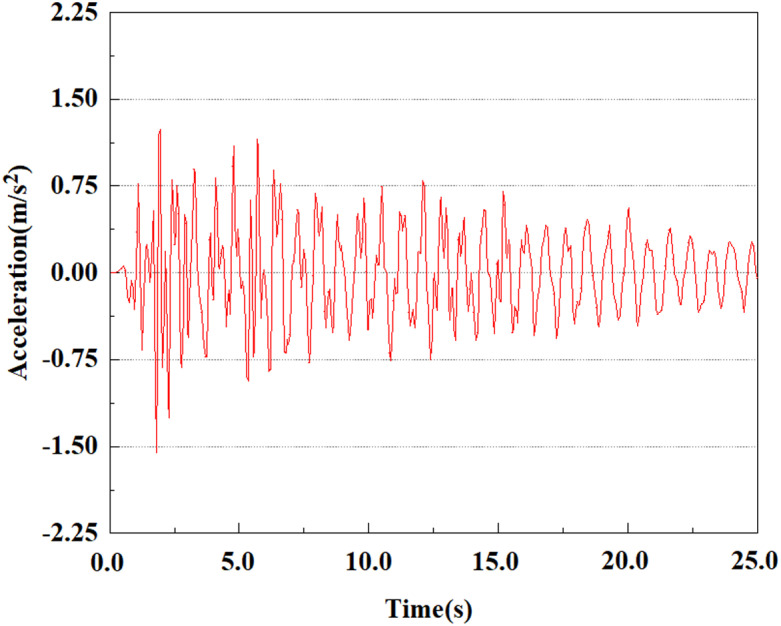
Changes in pile top acceleration under 0.05g EI wave magnitude at feature point 5.

**Fig 68:**
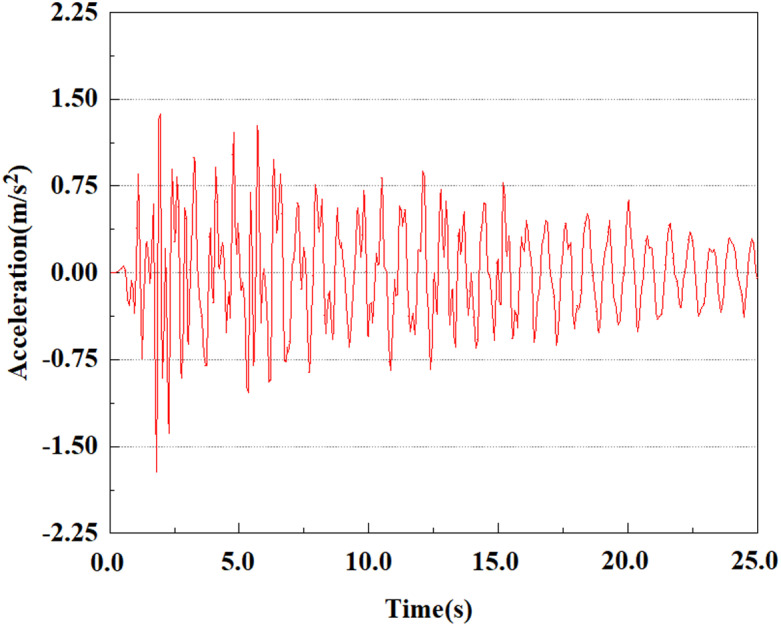
Changes in pile top acceleration under 0.25g EI wave magnitude at feature point 5.

**Fig 69:**
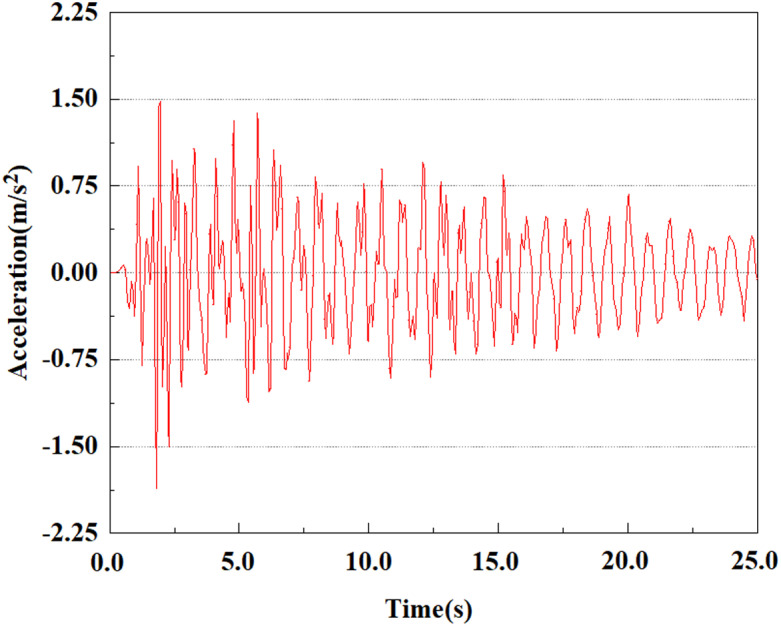
Changes in pile top acceleration under 0.50g EI wave magnitude at feature point 5.

From Figs 67–69, it is evident that the peak acceleration response varies with earthquake magnitude at various seismic intensities at the same pile position. At a magnitude of 0.05g, the peak acceleration at feature point 5 reached 1.62m/s ². At a magnitude of 0.25g, the peak acceleration at feature point 5 reached 1.72m/s ². At a magnitude of 0.5g, the peak acceleration at feature point 5 reached 1.85m/s ².

By analyzing the acceleration at the same location of the pile at varying seismic magnitudes, it can be drawn a conclusion that in the case of the same location of the pile characterization points, the value of acceleration response of the pile body grows as the earthquake magnitude increments, and the pile body’s peak acceleration lags behind the time of seismic wave vibration. Among the acceleration variations at varying locations of the pile body, it can be noted that the acceleration at the pile bottom stayed relatively stable with time, and the peak value was the smallest compared to other characteristic points. This is because the pile bottom bears a larger load, resulting in more significant constraints on the surrounding soil. This constraint causes the soil at the bottom of the pile to be compressed in the vertical direction, limiting its free deformation and thus applying greater restraining force. However, the vibration is relatively limited, so the vibration at the bottom of the pile is relatively stable. At the top of the pile, the peak acceleration is slightly larger as compared to the pile bottom, because the soil at the pile top is less constrained and the vibration is relatively free, resulting in a larger peak acceleration. The pile accelerates more quickly as the earthquake’s magnitude enhances. This is because the larger the earthquake magnitude, the stronger the intensity and energy of the seismic waves, the more intensely the pile body vibrates, the higher the dynamic stress that the seismic force applies to the pile body and the soil. Moreover, as the earthquake magnitude increases, the soil becomes less stable and less restraining to the pile body, leading to the acceleration of the pile body rising with the magnitude of the earthquake.

### 3.5 Analysis of changes in pile top displacement

**Fig 70:**
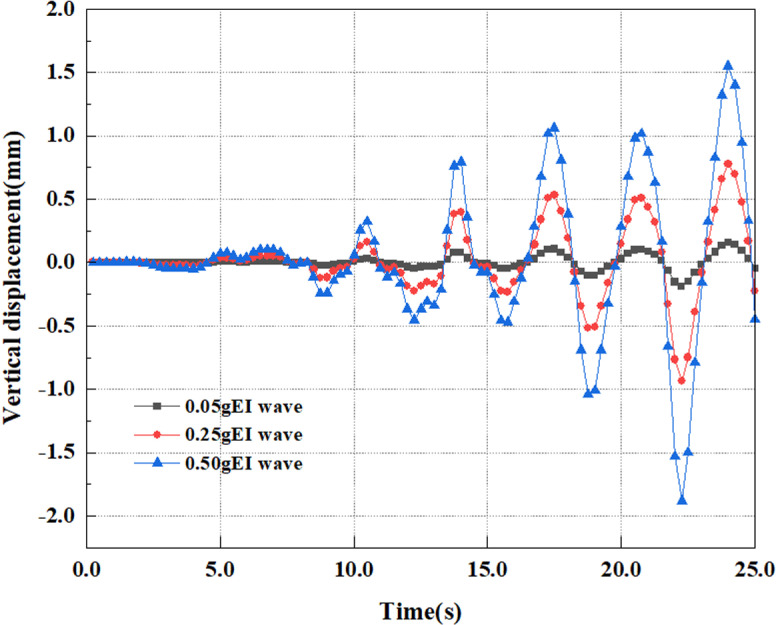
Changes in vertical displacement of pile top.

Determine the pile top’s deformation outcomes under various earthquake magnitude load scenarios. The fluctuation in the pile top’s vertical displacement under varying earthquake magnitudes is presented in Fig 70. Figure presents that the values and distributions of vertical displacements at the top of the pile varied under different earthquake magnitudes. Overall, under the same seismic time conditions, the vertical displacement at the pile top enhances as the seismic magnitude elevates, and as the seismic magnitude increases, the vertical displacement fluctuation in the latter half is also correspondingly severe.

**Fig 71:**
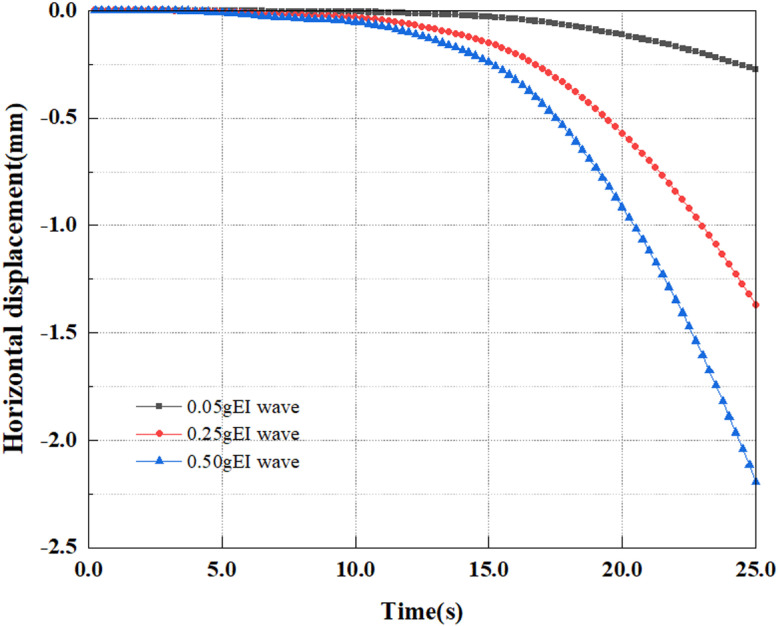
Changes in horizontal displacement of pile top.

Fig 71 illustrates the variance in the horizontal displacement of pile top under various earthquake magnitudes. Extract the deformation findings of the pile top under diverse earthquake magnitude load circumstances. From the graph, it can be seen that under different earthquake magnitudes, the horizontal displacement at the pile top shows a uniform variation, that is, with the application of dynamic loads over time, the horizontal displacement at the pile top will rise, and settlement at the pile top will also grow. Overall, under the same seismic time conditions, the pile top horizontal displacement grows as the magnitude elevates, and the growth rate of horizontal displacement progressively enhances in the latter half of the period as the magnitude increases. Due to the increase in seismic wave intensity, greater dynamic forces are applied to the pile body, such as horizontal inertial forces and seismic forces. This will cause the pile body to experience stronger vibrations, leading to an increase in the displacement of pile top. And the larger the magnitude of the earthquake, the more unstable the soil appears, resulting in a shift in pile support conditions and an incremental increase in pile displacements.

### 3.6 Analysis of pile axial force variation

**Fig 72:**
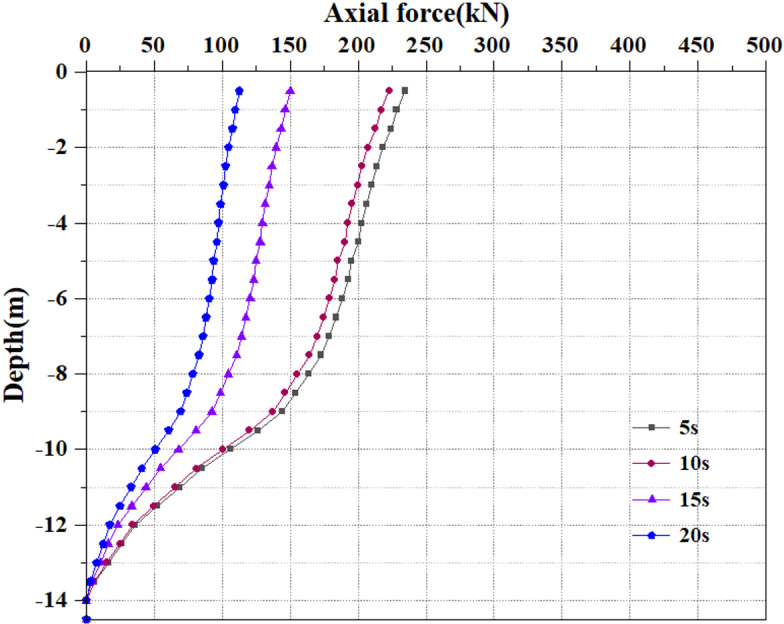
Changes in pile axial force under 0.05g EI wave earthquake magnitude.

**Fig 73:**
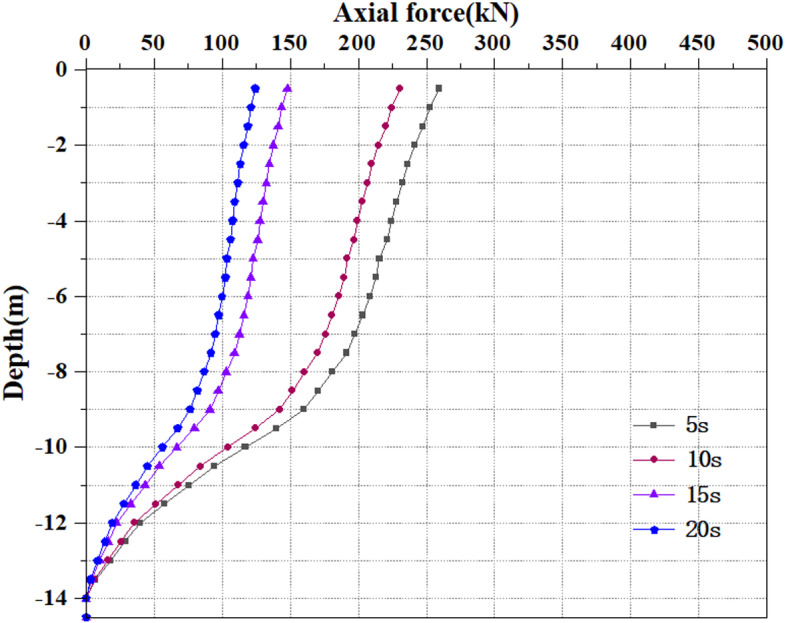
Changes in pile axial force under 0.25g EI wave earthquake magnitude.

**Fig 74:**
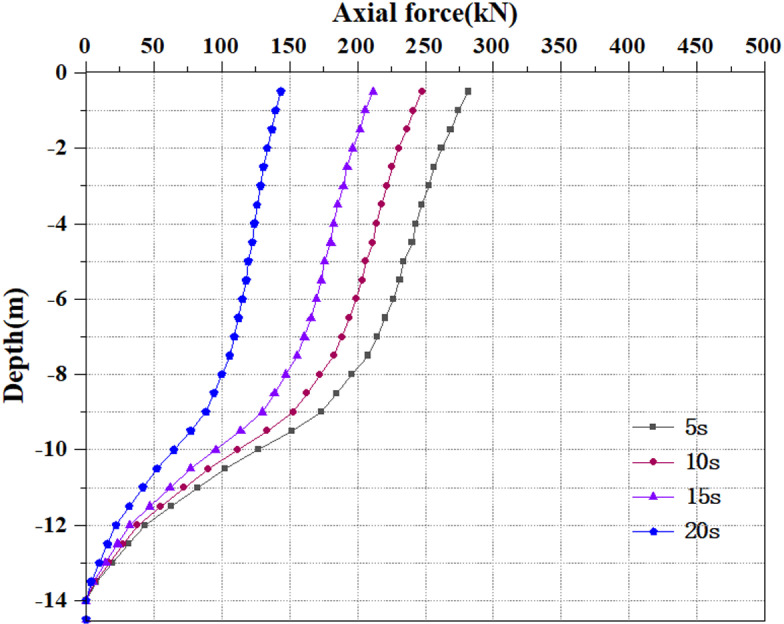
Changes in pile axial force under 0.50g EI wave earthquake magnitude.

Extract the pile axial force values under various seismic load scenarios. The relative displacement between the soil and the pile, or the axial force, and pile resistance are calculated based on the strain measured during static load testing [40]. Figs 72–74 show the pile axial force variation. The damping effect of soil is an important parameter, which is introduced into the dynamic response of soil to simulate energy loss and dissipate energy in seismic waves. The seismic wave of the model is applied to the soil surrounding the pile. Though the soil has some damping action, the vibration through the seismic wave, the seismic force will exert an upward force on the soil. At this time, the pile body will be compressed upwards by the soil, and there will inevitably be an upward sliding trend. At this time, friction force will inevitably be generated between the pile body and the soil, which results in the situation where there is still axial force on the pile body even if no load is applied above the pile top.

Figs 72–74 display the variation of axial force on the pile body under different earthquake magnitudes. The figure presents that under various earthquake magnitudes, the variation of axial force on the pile body shows a trend of larger upper part and smaller lower part. With the increase of dynamic load application time, the pile body is subjected to axial forces that progressively reduce from the top to the bottom. At the same time, as the earthquake magnitude increases, the axial force value of the pile body grows progressively. When the seismic wave has the peak acceleration of 0.05g, 0.25g and 0.50g, the maximum value of axial force at the horizon pile top are 237kN, 265kN and 281kN, respectively. Overall, the value and degree of variation of the pile axial force rise as the seismic magnitude enhances under the same seismic time circumstances. This is owing to an enhancement in seismic magnitude results in a rise in the intensity and energy of seismic waves, and the vibration amplitude of seismic waves increases, causing a rise in the seismic force on the pile body, thereby generating greater dynamic response to the pile body and soil. As the vibration time increases, the pile axial force reduces, which may be caused by the increase in vibration time. The soil and pile experience multiple reciprocating vibrations. During this process, the soil and pile gradually dissipate some seismic energy, causing settlement and damping effects, which will reduce the amplitude of the pile and the seismic force on the pile. Under earthquake action, the soil particles are in a suspended state, losing their bearing capacity, and the pile axial force progressively reduces.

**Fig 75:**
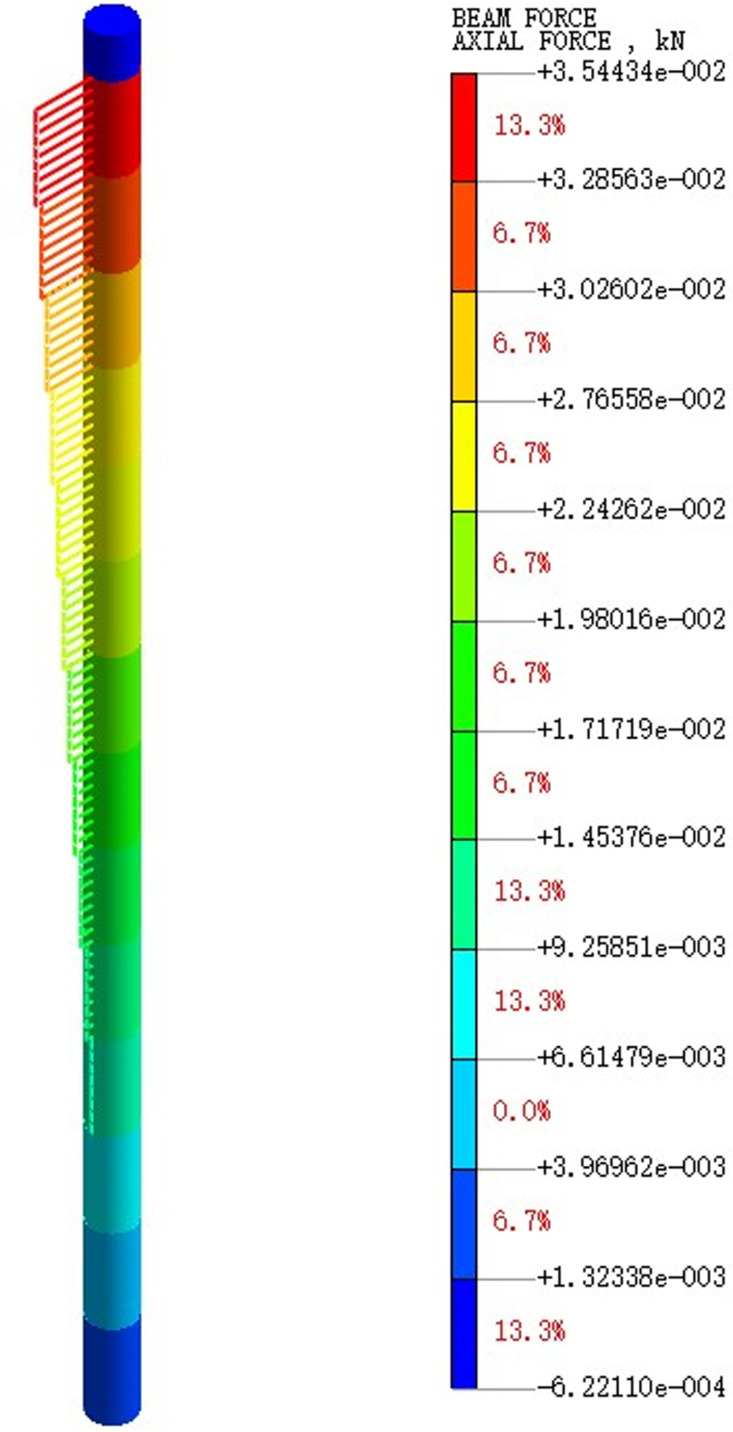
Cloud diagram of pile axial force variation under 0.05g EI wave earthquake magnitude.

**Fig 76:**
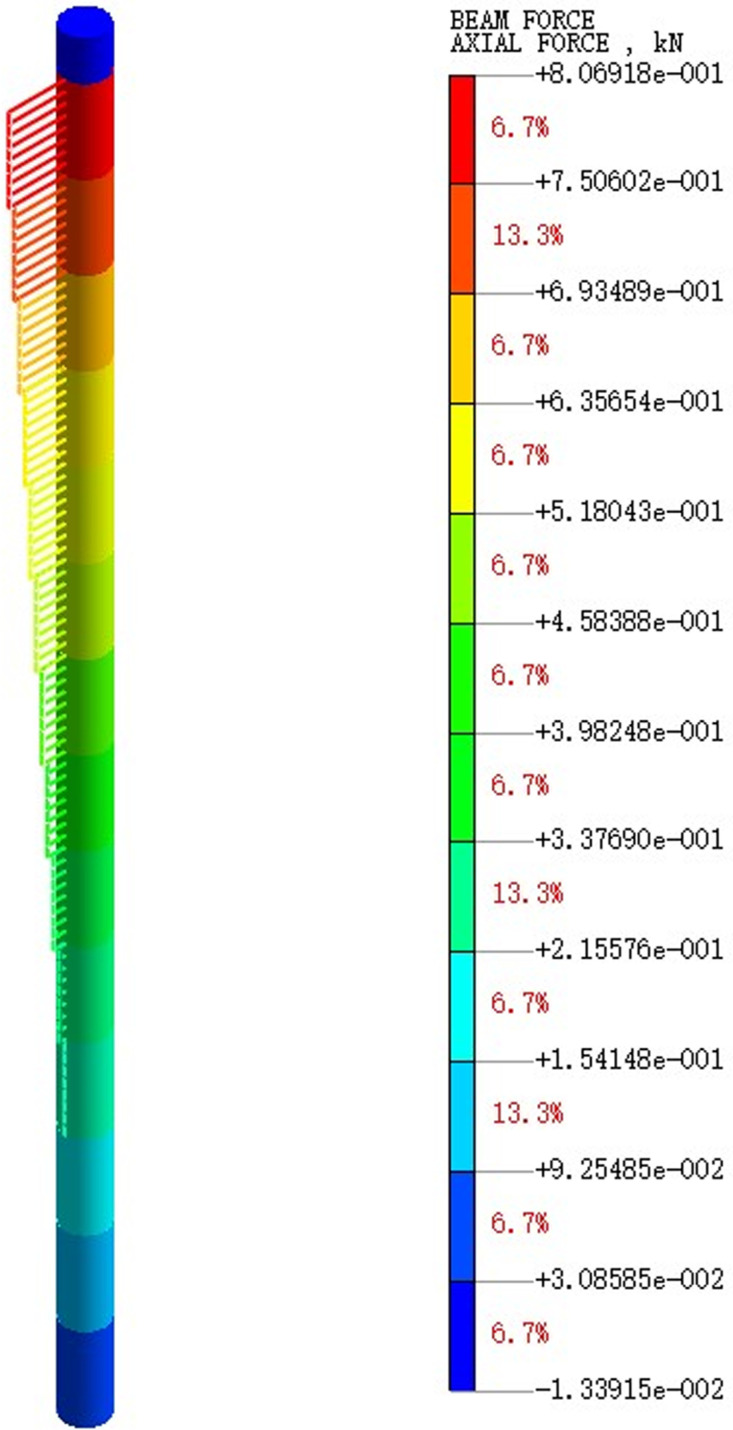
Cloud diagram of pile axial force variation under 0.25g EI wave earthquake magnitude.

**Fig 77:**
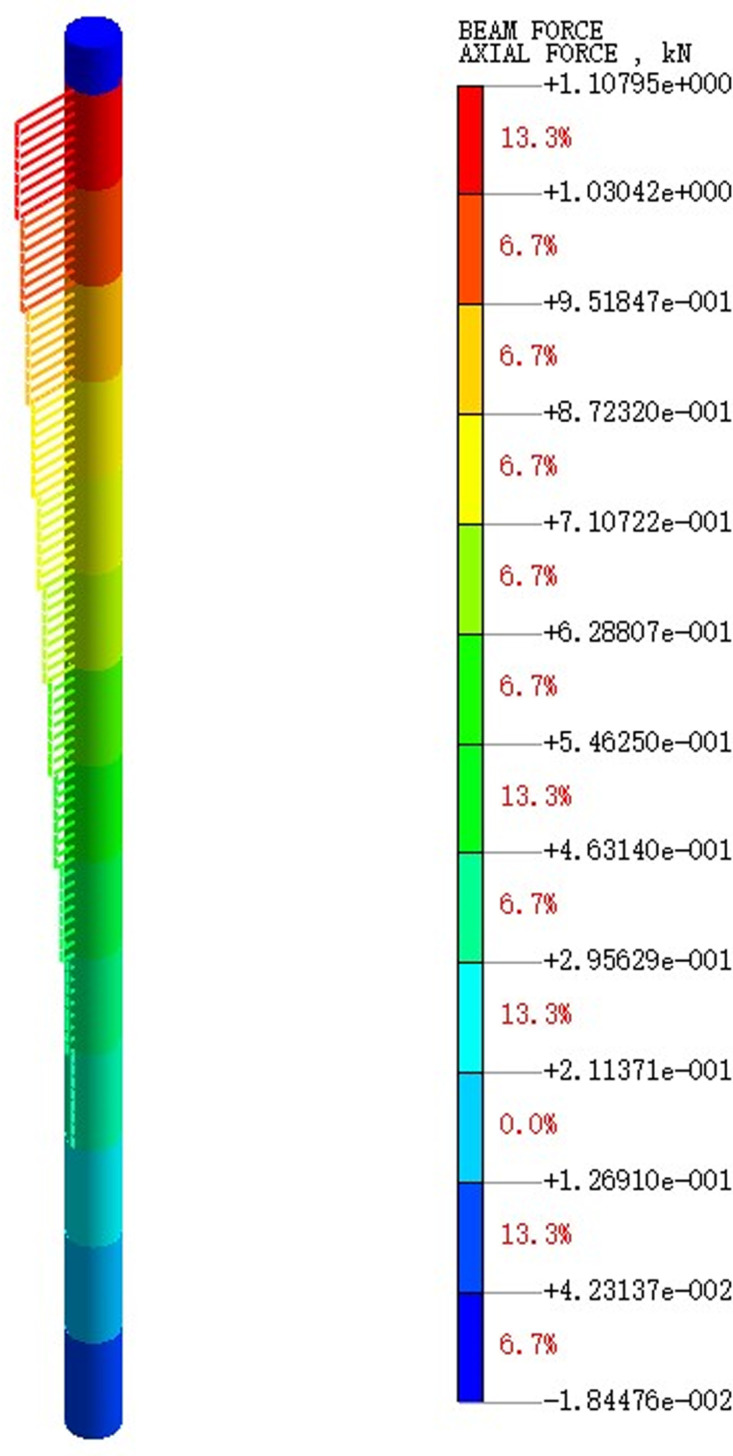
Cloud diagram of pile axial force variation under 0.50g EI wave earthquake magnitude.

Extract the pile axial force maps under varying earthquake magnitude load conditions. Figs 75–77 show the pile axial force variation under diverse earthquake magnitudes. The figure exhibits that under varying earthquake magnitudes, the variation of pile axial force is characterized by a larger upper part and a smaller lower part. This is due to the fact that the upper part of the pile is free end while the bottom of the pile is fixed in the bearing soil layer. With the vibration of seismic waves, particles in the soil will also vibrate and displacement will increase, which leads to a large amplitude of vibration at the pile top.

## 4 Conclusion

The problem of soil response under earthquake action is very complex. Dynamic response research has made major advances in the last few decades. However, due to the unpredictability and difficulty in reproducing earthquakes, scholars traditionally simulate the dynamic response between piles and soil under earthquake vibration through on-site scaled tests. In this paper, an effective finite element model is developed to examine the pile’s dynamic response under seismic action. It not only reflects the seismic process truthfully, but also establishes three different earthquake magnitude working conditions to realistically simulate the interaction between soil and piles under seismic action. With the seismic magnitude growing, the response of the pile-soil system becomes more severe. Nevertheless, there are still some shortcomings in this article. In practical engineering, the situation where the foundation is only a single pile is very rare, and foundation engineering is basically a group of piles. This work investigates the dynamic properties of soil mono-pile foundations under seismic action, which differs from actual engineering. In future research, the characteristics of group pile foundation under earthquake action should be further explored. In addition, considering the simplification of finite element calculations, this article treats the soil as a continuous, uniform, and isotropic medium for effective and accurate numerical simulation. However, in reality, the properties of soil are usually more complex, and their properties have a major effect on the seismic response of pile foundations. Therefore, in the future modeling process, the complex properties of soil need to be considered.

This article is based on the MIDAS GTS NX software to carry out numerical simulation analysis of pile soil system under seismic action. By varying the model amplitude, the dynamic analysis of pile-soil under seismic action has been performed and the bearing properties of the pile foundation under simulated actual environment were reproduced. Variations in soil pore pressure ratio and pore water pressure under earthquake action, as well as the changes in axial force, acceleration and displacement of pile body were obtained. The conclusions reached are as follows:

1. Under earthquake action, there is a huge top part and a little lower part to the pile axial force fluctuation, and the axial force grows gradually along with the dynamic load. Under the same vibration time, the maximum increase in pile state can be 40%. This indicates that the burial depth of the pile body and the geological strata will have an impact on the response of the pile body, playing an important role in engineering design and structural stability.
2. Under the same earthquake time conditions, as the earthquake magnitude grows, so does the vertical displacement at the pile top, while the horizontal displacement also elevates together with the earthquake magnitude. At the same vibration time, the vertical and horizontal displacements of the highest magnitude pile are approximately 9 times that of the lowest magnitude pile. This indicates that the magnitude of the earthquake is increasing, and the structure is subjected to more and more seismic forces.
3. As the magnitude of the earthquake increases, the pile acceleration becomes more severe, and the acceleration of pile incrementally elevates from the pile end to top. At different magnitudes, the acceleration at the top of the pile increases by an average of 20% compared to the acceleration at the bottom of the pile. At the same depth, the pile acceleration increases as the seismic intensity raise. This indicates that the properties of the soil and the depth of burial jointly affect the dynamic response of the pile body. This indicates that the dynamic response of the pile body is influenced by both soil properties and burial depth.
4. As the magnitude grows, the pore pressure ratio and pore water pressure also enlarge, and the time when they start to increase is getting earlier and earlier. This indicates that the intensity of seismic waves influences the stability of the soil’s internal structure, which needs to be considered in risk prevention in engineering. This indicates that the stability of the internal structure of the soil is affected by the intensity of seismic waves, and risk prevention should be considered in engineering construction.

## Supporting information

S1 supporting information https://doi.org/10.17605/OSF.IO/8UYTS
(XLSX)
